# Analysis of the SBP-SAT Stabilization for Finite Element Methods Part I: Linear Problems

**DOI:** 10.1007/s10915-020-01349-z

**Published:** 2020-11-03

**Authors:** R. Abgrall, J. Nordström, P. Öffner, S. Tokareva

**Affiliations:** 1 Institute of Mathematics, University of Zurich, Winterthurerstrasse 190, 8057 Zurich, Switzerland; 2 Department of Mathematics, Computational Mathematics, Linköping University, 581 83 Linköping, Sweden; 3 Department of Mathematics and Applied Mathematics, University of Johannesburg, P.O. Box 524, Auckland Park, 2006 South Africa; 4 Institute of Mathematics, Johannes Gutenberg-Universtiy, Staudingerweg 9, 55099 Mainz, Germany; 5 Theoretical Division, Applied Mathematics and Plasma Physics Group (T-5), Los Alamos National Laboratory, Los Alamos, NM 87545 USA

**Keywords:** Continuous Galerkin, Stability, Simultaneous approximation terms, Initial-boundary value problem, Hyperbolic conservation laws

## Abstract

In the hyperbolic community, discontinuous Galerkin (DG) approaches are mainly applied when finite element methods are considered. As the name suggested, the DG framework allows a discontinuity at the element interfaces, which seems for many researchers a favorable property in case of hyperbolic balance laws. On the contrary, continuous Galerkin methods appear to be unsuitable for hyperbolic problems and there exists still the perception that continuous Galerkin methods are notoriously unstable. To remedy this issue, stabilization terms are usually added and various formulations can be found in the literature. However, this perception is not true and the stabilization terms are unnecessary, in general. In this paper, we deal with this problem, but present a different approach. We use the boundary conditions to stabilize the scheme following a procedure that are frequently used in the finite difference community. Here, the main idea is to impose the boundary conditions weakly and specific boundary operators are constructed such that they guarantee stability. This approach has already been used in the discontinuous Galerkin framework, but here we apply it with a continuous Galerkin scheme. No internal dissipation is needed even if unstructured grids are used. Further, we point out that we do not need exact integration, it suffices if the quadrature rule and the norm in the differential operator are the same, such that the summation-by-parts property is fulfilled meaning that a discrete Gauss Theorem is valid. This contradicts the perception in the hyperbolic community that stability issues for pure Galerkin scheme exist. In numerical simulations, we verify our theoretical analysis.

## Introduction

In recent years, significant efforts have been made to construct and develop high-order methods for hyperbolic balance laws, and most of the methods are either based on finite difference (FD) or finite element (FE) approaches. In the FE framework, one favorable, if not the most favorable scheme, seems to be the discontinuous Galerkin (DG) method introduced by Reed and Hill [1] because of its stability properties [2–5]. Many modern DG formulations are based on summation-by-parts (SBP) operators and the recent stability proofs rely on the SBP property [5–10]. Even, if the SBP operators where originally defined in the FE framework, they have been transferred to FD methods [11] and have been further developed in the FD setting where they are now commonly used. They lead to stability following the steps of the continuous energy analysis [12–14]. Together with SBP operators, Simultaneous Approximation Terms (SATs) that impose the boundary conditions weakly are applied. The SBP-SAT technique is powerful and universally applicable as we will show in this paper. Another reason for the popularity of DG is that the numerical solution is allowed to have a discontinuity at the element boundaries, and, since non-linear hyperbolic problems are supporting shocks, this property is believed to be desirable. In addition and maybe most important, the DG methods leads to block diagonal mass matrices which are easy to invert. The difference between a DG approach and continuous Galerkin (CG), besides the structure of the mass matrix, is that in CG the approximated solution is forced to be continuous also over the element boundaries. This restriction is perceived to be quite strong also in terms of stability where the erroneous (as we will show) belief in the hyperbolic research community exists, that a pure CG scheme is unstable,^1^ and stabilization terms have to be applied to remedy this issue [15–17].

One may only speculate where this erroneous perception come from? In our opinion, one major reason could be that if one considers a pure Galerkin method using a linear Lagrange polynomial basis of order one, it can be shown that the method is equivalent to the 3-point central difference scheme. This scheme is not von Neumann stable [18] when periodic boundary conditions are considered. By switching the basis functions for instance to splines and / or some lumping technique, von Neumann stability can be proven, see [19, 20]. We want to point out that with the lumping technique as described in [20], one is able to re-write the Galerkin method to well-known finite difference schemes like Lax-Friedrichs or Lax-Wendroff schemes and at the end, a stable finite difference scheme.^2^ In addition, if one considers initial-boundary value problems, there also exist some preliminary stability results [21–23]. Here, the main idea is to switch the norms of the trial space and include the procedure at the boundary. However, these results seems forgotten in the hyperbolic community.

In this paper, we focus on the stability property of a pure Galerkin scheme, but follow a different approach. Our preliminary idea/thought is: If one considers the DG method with one element, the method is stable. There is nothing that says that the approximation space must be a broken polynomial space, the only thing that is needed is that the trial and test function must have some kind of regularity within the elements, so that the divergence theorem (or SBP techniques) can be applied. Continuity at the boundaries and regularity inside the elements due to the polynomial space are enough. No internal artificial dissipation is required and no special conditions on the grid structure, for instance cartesian grids, have to be assumed. Thus, for example unstructured triangular meshes can be applied. Hence, one can see a CG method as a DG one, with only one element (the union of the simplices) with an approximation space made of polynomials with continuity requirement between the simplices. Hence, what is the difference between these two approaches? The answer to this question points to the procedure at the boundary. In the stability proofs, the use of SATs is essential. In [13] diagonal norm stable CG SBP-SAT discretizations have previously been presented and further extended in [24, 25] where local projection stabilizations are applied to obtain entropy stable discretizations. The focus lies especially on the construction and investigation of diagonal norm SBP operators. Contrarily, in this work we focus on SAT and apply Galerkin schemes which fulfill the SBP property meaning that a discrete Gauss theorem is valid. We apply them with pure CG discretizations with dense norms and this is the topic of this paper where we show that no internal dissipation is needed in CG methods. We divide the paper as follows: In the second section, we shorty introduce the continuous Galerkin scheme which is used and investigated in the following. Next, we introduce and repeat the main idea of the SAT procedure from the FD framework and extend it to the Galerkin approach. We show that the determination of the boundary operators is essential. In Sect. 4, we focus on the eigenvalue analysis of the spatial operators and derive conditions from the continuous setting to build adequate boundary operators in the discrete framework. We give some recipes which will be used in Sect. 5 to support our analysis in numerical experiments. Finally, we conclude and discuss future work.

## Continuous Galerkin Scheme

In this section, we shortly introduce the pure continuous Galerkin scheme (CG) as it is also known in the literature [11, 16, 26]. We are interested in the numerical approximation of a hyperbolic problem

1 $$\begin{aligned} \frac{\partial U}{\partial t}+{\text {div}}\,f(U)=0 \end{aligned}$$

with suitable initial and boundary conditions. The domain $\Omega$ is split into subdomains $\Omega _h$ (e.g triangles/quads in two dimensions, tetrahedrons/hex in 3D). We denote by *K* the generic element of the mesh and by *h* the characteristic mesh size. Then, the degrees of freedom (DoFs) $\sigma$ are defined in each *K*: we have a set of linear forms acting on the set ${\mathbb {P}}^k$ of polynomials of degree *k* such that the linear mapping $q\in {\mathbb {P}}^k\longmapsto (\sigma _1(q),\ldots , \sigma _{|\sum _K|}(q))$ is one-to-one, where $|\sum _K|$ denotes the total number of DoFs in *K*. The set ${\mathcal {S}}$ denote the set of degrees of freedom in all elements. The solution *U* will be approximated by some element from the space ${\mathcal {V}}^h$ defined by

2 $$\begin{aligned} {\mathcal {V}}^h:={\bigoplus _{K} \left\{ U^h|_K \in {\mathbb {P}}^k \cap C^0(\Omega ) \right\} .} \end{aligned}$$

A linear combination of basis functions $\varphi _\sigma \in {\mathcal {V}}^h$ will be used to describe the numerical solution

3 $$\begin{aligned} U^h( x)=\sum _{K\in \Omega _h }\sum _{\sigma \in K} U_{\sigma }^h(t) \varphi _{\sigma }|_K( x), \quad \forall { x \in \Omega }. \end{aligned}$$

As basis functions we are working either with Lagrange interpolation where the degrees of freedom are associated to points in *K* or Bézier polynomials.

To start the discretisation, we apply a Galerkin approach and multiply with a test function $V^h$ and integrate over the domain. This gives

4 $$\begin{aligned} \int _{\Omega } (V^h)^T \frac{\partial U}{\partial t}{\mathrm {d}{x}}+ \int _{\Omega } (V^h)^T {\text {div}}\,f(U) {\mathrm {d}{x}} =0. \end{aligned}$$

Using the divergence theorem, we get

5 $$\begin{aligned} \int _{\Omega } (V^h)^T \frac{\partial U}{\partial t}{\mathrm {d}{x}}- \int _{\Omega } (\nabla V^h)^T f(U) {\mathrm {d}{x}} +\int _{\partial \Omega } \big (V^h\big )^Tf(U)\cdot \mathrm {n}\;d\gamma =0. \end{aligned}$$

By choosing $V^h=\varphi _{\sigma }$ for any $\sigma \in {\mathcal {S}}$, where we further assume for simplicity that our basis functions vanishes at the physical boundaries, we obtain with (3) a system of equations:

6 $$\begin{aligned} \sum _{K\in \Omega _h }\sum _{\sigma ' \in K} \bigg ( \frac{\partial U_{\sigma '}^h(t)}{\partial t} \int _K\varphi _{\sigma '}(x) \varphi _\sigma (x) {\mathrm {d}{x}} - \int _{K} \nabla \varphi _{\sigma '}(x)\; f(U^h) {\mathrm {d}{x}}\bigg ) =0. \end{aligned}$$

Our approach makes (6) fully explicit with special focus on the basis functions. The contributions from the boundary integral are removed due to the assumption that $\varphi |_{\partial \Omega }=0$. Our motivation for this simplification is driven by the fact that we want to avoid a discussion about boundary conditions in this part which will be the content of the followings sections. The internal contributions cancel out through the different signs in the normals. In practice, we compute (6) with a quadrature rule:

$$\begin{aligned} \sum _{K\in \Omega _h }\sum _{\sigma ' \in K} \bigg ( \frac{\partial U_{\sigma '}^h(t)}{\partial t} \; \oint _K\varphi _{\sigma '}(x) \varphi _\sigma (x) {\mathrm {d}{x}} - \oint _{K} \nabla \varphi _{\sigma '}(x)\; f(U^h) {\mathrm {d}{x}} \bigg ) =0, \end{aligned}$$

where $\oint$ represents the quadrature rules for the volume and surface integrals.

In this paper, we are considering linear problems, i.e. the flux is linear in *U*, but may depend on the spatial coordinate. In all the numerical experiences, we will make the spatial dependency simple enough (i.e. typically polynomial in *x*), so that it will always be possible to find a standard quadrature formula and obtain accurate approximations for the integrals. Note, if the quadrature rule is accurate enough, (6) can be exactly reproduced for linear problems with constant coefficients.

Using a matrix formulation, we obtain the classical FE formulation:

7 $$\begin{aligned} {\underline{\underline{M}}}\,\frac{\partial }{\partial t}\underline{U}^h +{\underline{\underline{{\mathbf {F}}}}}\,=0, \end{aligned}$$

where $\underline{U}^h$ denotes the vector of degrees of freedom, ${\underline{\underline{{\mathbf {F}}}}}\,$ is the approximation of ${\text {div}}\,f$ and ${\underline{\underline{M}}}\,$ is a mass matrix.^3^ For continuous elements, this matrix is sparse but not block diagonal, contrary to the situation for the discontinuous Galerkin methods. Due to the rumor/perception in the hyperbolic community that a pure Galerkin scheme suffers from stability issues for hyperbolic problems, it is common to add stabilization terms to the scheme as for example in [17]. However, as previously mentioned we take a different approach in this paper and will renounce these classical stabilization techniques. In order to do this, we need more known results from the literature, which we will briefly repeat here.

## Weak Boundary Conditions

To preserve the structure of the SBP operators, and facilitate proofs of stability, weak boundary conditions are preferable over strong one’s.

### SATs in SBP-FD Framework

To implement the boundary conditions weakly using simultaneous approximation terms (SATs) is nowadays standard in the FD community and has been developed there. Together with summation-by-parts (SBP) operators it provides a powerful tool for proofs of semidiscrete ($L_2$) stability of linear problems by the energy method, see [12, 14, 28] for details.

Here, we present a short introductory example of the SBP-SAT technique as it is presented in [14, 27]. Consider the linear advection equation

8 $$\begin{aligned} \begin{aligned} \frac{\partial u}{\partial t}+a \frac{\partial u}{\partial x}&=0, \quad 0\le x\le 1, \quad t>0,\\ u(x,0)&=u_{in}(x),\\ u(x,t)&=b(x,t)\quad \text { for inflow boundary}, \end{aligned} \end{aligned}$$

where $u_{in} \in$ is the initial condition and *b* is the known boundary data that is only defined on the inflow part of $\partial [0,1]=\{0,1\}$. In other words, if $a>0$, then *b* is only set for $x=0$, and if $a<0$, this will be for $x=1$ only.

To explain the semi-discretisation of (8), we consider the discrete grid $\underline{x}=(x_0, \ldots , x_N)^T$, with the ordering of nodes $x_0=0<\cdots <x_N=1$. Furthermore, the spatial derivative of a function $\phi$ is approximated through a discrete derivate matrix ${\underline{\underline{D}}}\,$, i.e. $\phi _x\approx {\underline{\underline{D}}}\, \underline{\phi }$ with $\underline{\phi }=(\phi (x_0),\; u_1,\;, \ldots , \phi (x_N))^T$. It is defined by

#### Definition 3.1

(SBP operators) An operator ${\underline{\underline{D}}}\,$ is a *p*-th order accurate approximation of the first derivative on SBP form if

1. $${\underline{\underline{D}}}\,\underline{x}^j={\underline{\underline{M}}}\,^{-1}{\underline{\underline{Q}}}\, \underline{x}^j= j \underline{x}^{j-1}, \; j \in [0,p] $$ with $$\underline{x}^j= (x_0^j, \ldots , x_N^j)^T$$,
2. ${\underline{\underline{M}}}\,$ is a symmetric positive definite matrix,
3. $${\underline{\underline{Q}}}\,+ {\underline{\underline{Q}}}\,^T={\underline{\underline{B}}}\,=\mathop {{\mathrm {diag}}}(-1,0, \ldots ,0,1)$$ holds.

Now, a semi-discretisation of (8) is given in terms of SBP operators as

9 $$\begin{aligned} \begin{aligned} \frac{\partial \underline{u}}{\partial t}+a{\underline{\underline{D}}}\,\underline{u}&={\underline{\underline{M}}}\,^{-1}\underline{{\mathbb {S}}} , \quad t>0,\\ \underline{u}(0)&=\underline{u}_{in}, \end{aligned} \end{aligned}$$

where $\underline{u}=(u_0,\; u_1,\;, \ldots , u_N(t))^T$ are the coefficients of *u* and similarly for $\underline{u}_{in}$. The coefficients are evaluated on the nodal values, i.e. the grid points, and are used to express the numerical solution (3). Translating this into the FE framework, they correspond to the coefficients for the degrees of freedoms. The symmetric positive definite matrix ${\underline{\underline{M}}}\,$ approximates the usual $L^2$ scalar product. Together with condition 3. from Definition 3.1, we mimic integration by parts discretely, i.e.

10 $$\begin{aligned} \begin{aligned} v(1)u(1)-u(0)v(0)=&\int _0^1 u(x) v'(x) {\mathrm {d}{x}} + \int _0^1 u'(x) v(x) {\mathrm {d}{x}} \\ \approx&\underline{u} {\underline{\underline{M}}}\,{\underline{\underline{D}}}\,\underline{v} +\underline{u} {\underline{\underline{D}}}\,^T{\underline{\underline{M}}}\, \underline{v}=\underline{u} {\underline{\underline{B}}}\,\underline{v}. \end{aligned} \end{aligned}$$

In (10), we have for smooth functions *u*

11 $$\begin{aligned} {\underline{\underline{D}}}\,\underline{u}\approx \frac{\partial }{\partial x} u \text { and } ||\underline{u}||_{{\underline{\underline{M}}}\,}^2:= \underline{u}^T{\underline{\underline{M}}}\,\underline{u} \approx \int _{0}^{1} u^2(x){\mathrm {d}{x}}. \end{aligned}$$

Instead of having an extra equation on the boundary like in (8), the boundary condition is enforced weakly by the term $\underline{{\mathbb {S}}}=({\mathbb {S}}_0,0,\ldots , {\mathbb {S}}_N)^T$ which is called the SAT. We demonstrate how it should be selected to guarantee stability for (9).

#### Definition 3.2

The scheme (9) is called strongly energy stable if

12 $$\begin{aligned} ||\underline{u}(t)||_{{\underline{\underline{M}}}\,}^2\le K(t)\left( ||\underline{u}_{in}||_{{\underline{\underline{M}}}\,}^2+ \max _{t_1\in [0,t]} {|b(t_1)|}^2 \right) \end{aligned}$$

holds. The term *K*(*t*) is bounded for any finite *t* and independent from $u_{in}$, *b* and the mesh.

#### Remark 3.3

The Definition 3.2 is formulated in terms of the initial value problem (8) where only one boundary term is fixed. If an additional forcing function is considered at the right hand side of (8), we include the maximum of this function in (12) in the spirit of *b*, for details see [14].

As established in [29], we can prove the following:

#### Proposition 3.4

Let ${\underline{\underline{D}}}\,={\underline{\underline{M}}}\,^{-1}{\underline{\underline{Q}}}\,$ be an SBP operator defined in 3.1 with ${\underline{\underline{Q}}}\,$ fulfilling

13 $$\begin{aligned} {\underline{\underline{Q}}}\,+{\underline{\underline{Q}}}\,^T={\underline{\underline{B}}}\,=\mathop {{\mathrm {diag}}}(-1,0, \ldots ,0,1). \end{aligned}$$

Let $a^+=\max \{a,0\}$ and $a^-=\min \{a,0\}$, $b_0=b(0,t)$ and $b_N=b(1,t)$. If ${\mathbb {S}}_0=\tau _0 a^+ (u_0-b_0)$ and ${\mathbb {S}}_N=\tau a_N^- (u_N-b_N)$ with $\tau _0, \tau _N < - \frac{1}{2}$, then the scheme (9) is strongly energy stable.

#### Proof

Multiplying (9) with $\underline{u}^T{\underline{\underline{M}}}\,$ yields

14 $$\begin{aligned} \underline{u}^T{\underline{\underline{M}}}\, \frac{\partial }{\partial t} \underline{u}+a\underline{u}^T{\underline{\underline{M}}}\,{\underline{\underline{D}}}\,\underline{u}=\underline{u}^T\underline{{\mathbb {S}}}. \end{aligned}$$

Transposing (14) and adding both equations together leads to

$$\begin{aligned} \frac{\mathrm{d}}{\mathrm{d t }} ||\underline{u}||_{{\underline{\underline{M}}}\,}^2= \underline{u}^T{\underline{\underline{M}}}\, \frac{\partial }{\partial t} \underline{u} + \frac{\partial }{\partial t}\underline{u}^T {\underline{\underline{M}}}\,\underline{u}=-a \underline{u}^T ({\underline{\underline{Q}}}\,+{\underline{\underline{Q}}}\,^T) \underline{u}+2\underline{u}^T\underline{{\mathbb {S}}}. \end{aligned}$$

Further, we obtain from (13)

$$\begin{aligned} \frac{\mathrm{d}}{\mathrm{d t }} ||\underline{u}||_{{\underline{\underline{M}}}\,}^2= \bigg (a u_0^2 +2a^+\tau u_0(u_0-b_0)\bigg ) -\bigg (au_{N}^2 -2a^-\tau u_N(u_N-b_N)\bigg ). \end{aligned}$$

If $\tau _0, \tau _N<- \frac{1}{2}$, we find

$$\begin{aligned} \frac{\mathrm{d}}{\mathrm{d t }} ||\underline{u}||_{{\underline{\underline{M}}}\,}^2\le -\frac{a^+\tau ^2}{(1+2\tau )} b_0^2+\frac{a^-\tau ^2}{(1+2\tau )} b_N^2. \end{aligned}$$

$$\square $$

This shows that the boundary operator ${\mathbb {S}}$ can be chosen in such way that it guarantees stability for the SBP-SAT approximation of (8). Next, we will apply this technique in the Galerkin framework.

### SATs in the Galerkin-Framework

Instead of working with SBP-FD framework we consider now a Galerkin approach for the approximation of (8). In [5, 6], it is shown that the specific DG schemes satisfies a discrete summation-by-parts (SBP) property and can be interpreted as SBP-SAT schemes with a diagonal mass matrix. In this context, one speaks about the **discontinuous Galerkin spectral element method** (DGSEM). Here, we consider nodal continuous Galerkin methods and focus on stability conditions in this context. As we described already in Sect. 2, the difference between the continuous and discontinuous Galerkin approach is the solution space (2) and the structure of the mass matrix (7) which is not block diagonal in CG. However, in the following we consider only Galerkin approaches which fulfill the SBP property meaning that a discrete Gauss theorem is valid. The approach with SAT terms can still be used to ensure stability also in case of CG but one has to be precise, as we will explain in the following. Let us step back to the proof of Proposition 3.4 and have a closer look. Essential in the proof is condition (13). Let us focus on this condition for a Galerkin discretisation of (8) as described also in [27]. We approximate equation (8) now with $u^h(x, t)=\sum \nolimits _{j=0}^N u^h_j(t)\varphi _j(x)$ where $\varphi _j$ are basis functions and $u^h_j$ are the coefficients. First, we consider the problem without including the boundary conditions. Let us assume that $\varphi _j$ are Lagrange polynomials where the degrees of freedoms are associated to points in the interval. Introducing the scalar product

$$\begin{aligned} \left\langle u,v\right\rangle =\int _I u(x)v(x)\; {\mathrm {d}{x}} , \end{aligned}$$

let us consider the variational formulation of the advection equation (8) with test function $\varphi _i$. We insert the approximation and get

$$\begin{aligned} \begin{aligned} \left\langle \frac{\partial }{\partial t} u^h(t,x), \varphi _i(x) \right\rangle + \left\langle a \frac{\partial }{\partial x}u^h(t,x), \varphi _i (x)\right\rangle&=0, \quad \forall i=0, \ldots , N, \end{aligned} \end{aligned}$$

i.e.

$$\begin{aligned} \begin{aligned} \int _I \sum _{j=0}^N ( \frac{\partial }{\partial t} u^h_j(t)) \varphi _j(x)\varphi _i(x) {\mathrm {d}{x}}+a \int _I \sum _{j=0}^Nu^h_j(t)( \frac{\partial }{\partial x}\varphi _j(x) ) \varphi _i(x) {\mathrm {d}{x}}&=0. \end{aligned} \end{aligned}$$

Finally, we get

15 $$\begin{aligned} \begin{aligned} \sum _{j=0}^{N} M_{i,j}( \frac{\partial }{\partial t}u^h_j(t) ) + a\sum _{j=0}^{N} Q_{i,j} u_j^h(t)&=0\\ \end{aligned} \end{aligned}$$

with

16 $$\begin{aligned} M_{i,j}= \int _I \varphi _j(x)\varphi _i(x) {\mathrm {d}{x}} \quad \text { and } \quad Q_{i,j}= \int _I \left( \frac{\partial }{\partial x} \varphi _j(x)\right) \varphi _i(x) {\mathrm {d}{x}}. \end{aligned}$$

In matrix formulation (15) can be written

$$\begin{aligned} {\underline{\underline{M}}}\,\frac{\partial }{\partial t} \underline{u} +a{\underline{\underline{Q}}}\,\underline{u}=0 \end{aligned}$$

as described in [27]. Let us check (13). We consider

17 $$\begin{aligned} \begin{aligned} Q_{i,j}+Q_{i,j}^T&= \int _I \left( \frac{\partial }{\partial x} \varphi _j(x)\right) \varphi _i(x) {\mathrm {d}{x}} +\int _I\left( \frac{\partial }{\partial x} \varphi _i(x)\right) \varphi _j(x) {\mathrm {d}{x}} \\&= \int _I \frac{\partial }{\partial x} \left( \varphi _j(x) \varphi _i(x) \right) {\mathrm {d}{x}} = \varphi _i(x)\varphi _j(x)|_{0}^1\\&=\varphi _i(1)\varphi _j(1)-\varphi _i(0)\varphi _j(0) \quad \forall i,j =0, \ldots , N. \end{aligned} \end{aligned}$$

If the boundaries are included in the set of degrees of freedom, then we obtain

$$\begin{aligned} \varphi _i(1)\varphi _j(1)-\varphi _i(0)\varphi _j(0)={\left\{ \begin{array}{ll} 1 &{}\text { for } i=j=N,\\ -1 &{}\text { for } i=j=0,\\ 0 &{} \text{ elsewhere }. \end{array}\right. } \end{aligned}$$

Up to this point exact integrals are considered but the same steps are valid if a quadrature rule is applied such that (13) is satisfied and (17) is mimicked on the discrete level. This is ensured if the SBP property is fulfilled. Note that in this paper we only consider Galerkin schemes which guarantee this property. However, if we include a weak boundary condition similar to (9), we obtain the semidiscrete scheme

18 $$\begin{aligned} \begin{aligned} \sum _{j=0}^{N} M_{i,j}( \frac{\partial }{\partial t}u^h_j(t) ) + a\sum _{j=0}^{N} Q_{i,j} u_j^h(t)&={\mathbb {S}}\\ \end{aligned} \end{aligned}$$

with the SAT term given by

19 $$\begin{aligned} {\mathbb {S}}:=\tau a^+ (u_0-b_0)\delta _{x=0}+\tau a^- (u_N-b_N)\delta _{x=x_N=1}. \end{aligned}$$

By following the steps from the proof of Proposition 3.4, we can prove:

#### Proposition 3.5

If the Galerkin method (18) is applied to solve (8) with ${\mathbb {S}}$ given by (19) and $\tau {<} - \frac{1}{2}$, it is strongly energy stable.

#### Proof

The weak formulation of the problem reads:

$$\begin{aligned} \begin{aligned}&\left\langle \frac{\partial }{\partial t} u^h(t,x), \varphi _i(x) \right\rangle + \left\langle a \frac{\partial }{\partial x}u^h(t,x), \varphi _i (x)\right\rangle \\&\quad = \tau a^+ (u^h(0,t)-b_0(t))\varphi _i(0) +\tau a^- (u^h(1,t)-b_N)\varphi _i(1), \end{aligned} \end{aligned}$$

for all $i=0,\ldots , N$, where for simplicity, we consider the case $a>0$. The SAT techniques adds a penalty term into the approximation (18) on the right side. We focus now on the energy. Therefore, we multiply also with $u^h$ instead of $\varphi _i$ and rearrange the terms. We obtain for the semi-discretization (18):

$$\begin{aligned} \sum _{i,j,=0}^{N} M_{i,j}\left( \frac{\partial }{\partial t}u^h_j(t) \right) u^h_i(t) + a\sum _{i,j=0}^{N} Q_{i,j} u_j^h(t)u_i^h(t) = a\tau u^h_0(t)(u^h_0(t)-b_0(t)), \end{aligned}$$

where we used the fact that $u^h(t,0)=\sum _{i=0}^N u_i^h(t)\varphi _i(0)=u_0^h(t)$ is valid. By following the steps of the proof of Proposition 3.4 and using (17) we get the final result. $\square$

In the derivation above, we restricted ourselves to one-dimensional problems using Lagrange interpolations. Nevertheless, this shows that a continuous Galerkin method is stable if the boundary condition is enforced by a proper penalty term. For the general FE semi-discretization of (7), the procedure is similar and straightforward. Without loss of generality, it is enough to consider homogeneous boundary conditions and for a general linear problem (scalar or systems) the formulation (7) can be written with penalty terms as

20 $$\begin{aligned} {\underline{\underline{M}}}\, \frac{\partial }{\partial t}\underline{\mathbf{U}}^h+{\underline{\underline{Q_1}}}\,\ \underline{{\mathbf {U}}}^h= {\Pi } ( \underline{{\mathbf {U}}}^h ), \end{aligned}$$

where ${\Pi }$ is the boundary operator which includes the boundary conditions. This operator can be expressed in the discretization by a matrix vector multiplication. With a slight of abuse of notation, we denote this boundary matrix with ${\underline{\underline{\Pi }}}\,$ and it is usually sparse. Further, ${\underline{\underline{Q_1}}}\,$ represent the spatial operator and ${\underline{\underline{Q_1}}}\, +{\underline{\underline{Q_1}}}\,^T$ has only contributions on the boundaries. Then, we can prove.

#### Theorem 3.6

Apply the general FE semidiscretisation (20) together with the SAT approach to a linear equation and let the mass matrix ${\underline{\underline{M}}}\,$ of (20) be symmetric and positive definite. If the boundary operator ${\underline{\underline{\Pi }}}\,$ together with the discretization ${\underline{\underline{Q_1}}}\,$ can be chosen such that

21 $$\begin{aligned} ({\underline{\underline{\Pi }}}\,-{\underline{\underline{Q_1}}}\,)+ \left( {\underline{\underline{\Pi }}}\,-{\underline{\underline{Q_1}}}\, \right) ^T \end{aligned}$$

is negative semi-definite, then the scheme is energy stable.

#### Proof

We use the energy approach and multiply our discretization with ${\mathbf {U}}^h$ instead of $\varphi _i$ and add the transposed equation using ${\underline{\underline{M}}}\,^T={\underline{\underline{M}}}\,$. We obtain

$$\begin{aligned} \dfrac{d}{d t} || \underline{{\mathbf {U}}}^h||^2_{{\underline{\underline{M}}}\,} =\underline{{\mathbf {U}}}^{h,T} \left( ({\underline{\underline{\Pi }}}\,-{\underline{\underline{Q_1}}}\,)+ \left( {\underline{\underline{\Pi }}}\,-{\underline{\underline{Q_1}}}\,\right) ^T \right) \underline{{\mathbf {U}}}^{h} \le 0. \end{aligned}$$

$$\square $$

#### Remark 3.7

This theorem yields directly conditions when a FE method is stable, or not. If the matrix (21) has positive eigenvalues $\{\lambda _i\}$, stabilization terms have to be added. **However, no internal stabilization terms are necessary when** (21) ** is negative semi-definite.** Therefore, a number of requirements are needed. The distribution of the residuals attached to the degrees of freedom should be done in an “intelligent” way e.g. if we consider triangle elements and polynomial order $p=1$, we set the DoFs in every edge and not all of them on one face. Further, the chosen quadrature rule in the numerical integration has to be the same as in the differential operators. This means that the applied quadrature rule to calculate the mass matrix should be the same as the used one to determine the differential operators, such that SBP property is fulfilled meaning that a discrete Gauss Theorem is valid. In the numerical test, we will present an example of what happens if the chosen quadrature rules disregard this. Furthermore, in case of a non-conservative formulation of the hyperbolic problem or in case of variable coefficients a skew-symmetric/split formulation should be applied in the way described in [27, 30, 31]. In the one-dimensional setting, we obtain in the continuous case

$$\begin{aligned} \partial _x (au) =\alpha \partial _x(au) + (1-\alpha ) \left( u (\partial _x a) + a(\partial _x u)\right) \end{aligned}$$

with $\alpha =0.5$ and the implementation has to mimic this behavior.

If the implementation of the continuous Galerkin method is done in such way that the matrix (21) is negative semi-definite, then the method is stable only through our boundary procedure. In our opinion, this is **contrary** to common belief about continuous Galerkin methods for hyperbolic problems. The only stabilizing factor needed is a proper implementation of boundary conditions. For the linear scalar case, the proof is given in Proposition 3.5. In the following, we will extend this theory to more general cases.

#### Remark 3.8

(Weak Boundary Conditions in Galerkin Methods for Hyperbolic Problems) The weak formulation of the boundary condition is not done for the first time to analyze stability properties in continuous Galerkin methods. As already mentioned in the introduction, in [21–23] the authors have included the procedure at the boundary in their stability analysis where the main idea is to switch the norm of the trial space to prove stability.

Further, in [32] the authors have compared weak and strong implementation of the boundary conditions for incompressible Navier Stokes when the boundary condition is discontinuous and $C^0$ approximations are used. Here, non-physical oscillations are arising and by switching to the weak implementation, the authors have been able solve this issue. However, as a baseline schemes they have always supposed a stabilized Galerkin methods like SUPG in their theoretical considerations and applied it in their numerical examples. They have not imposed the weak boundary condition to stabilize their baseline scheme, but to cancel out these oscillations. In Nitsche’s method [33] for elliptic and parabolic problems, the boundary conditions are also imposed weakly. Here, the theoretical analysis is based on the bilinear from. However, further extensions of this method can be found and also a comparison to several DG methods. For an introduction and some historical remark, we strongly recommend [2] for more details. Finally, in DG methods it is common to impose boundary conditions weakly in hyperbolic problems and a detailed analysis for Friedrichs’ system [34] can be found in [35] which is oriented more on the variational formulation.

Finally, we want to point out again that the purpose of this paper is to demonstrate that no further internal dissipation is needed if the boundary conditions are implemented correctly. In addition, our analysis holds also if we apply unstructured grids as demonstrated in the numerics section below.

## Estimation of the SAT-Boundary Operator

As described before, a proper implementation of the boundary condition is essential for stability. Here, we give a recipe for how these SAT boundary operators can be chosen to get a stable CG scheme for different types of problems. First, we consider a scalar equation in 2D and transfer the eigenvalue analysis for the spatial operator from the continuous to the discrete setting. Then, we extend our investigation to two dimensional systems. Using again the continuous setting, we develop estimates for $\Pi$ and transfer the results to the finite element framework. We apply them later in the numerical section.

### Eigenvalue Analysis

We derive conditions on the boundary operators and perform an eigenvalue analysis to get an energy estimate in the continuous setting. Next, the results are transformed to the discrete framework to guarantee stability of the discrete scheme.

#### The Scalar Case

#### Continuous Setting

Consider the initial boundary value problem

22 $$\begin{aligned} \begin{aligned}&\frac{\partial }{\partial t}u+a \frac{\partial }{\partial x} u+b \frac{\partial }{\partial y}u =0&x\in \Omega , \quad&t>0, \\&Bu=g&x\in \partial \Omega , \quad&t>0,\\&u=f&x\in \Omega ,\quad&t=0 \end{aligned} \end{aligned}$$

in the spatial domain $\Omega \subset {\mathbb {R}}^2$. Further, $a,b \in {\mathbb {R}}$, the function *f* describes the initial condition, *B* represents the boundary operator and the function *g* the boundary data. Without loss of generality, it is enough to consider homogeneous boundary conditions and we consider the spatial operator

23 $$\begin{aligned} \begin{aligned} Du&:= \left( a\frac{\partial }{\partial x} +b\frac{\partial }{\partial y}\right) u, \\ \end{aligned} \end{aligned}$$

considered in the subspace of functions for which $Bu=0$. This operator will be dissipative if $\left\langle u,Du\right\rangle >0$. Using the Gauss-Green theorem, we obtain

24 $$\begin{aligned} \left\langle u,Du\right\rangle =\int _\Omega uDu \; {\mathrm {d}{\Omega }}= \int _{\partial \Omega }\frac{a}{2}u^2 {\mathrm {d}{y}} -\frac{b}{2}u^2{\mathrm {d}{x}} = {\frac{1}{2}} \int _{\partial \Omega } \underbrace{ (a,b) \cdot \mathrm {n}}_{:=a_n} u^2 {\mathrm {d}{s}}. \end{aligned}$$

The operator is hence dissipative if $\int _{\partial \Omega } a_n u^2{\mathrm {d}{s}}>0.$ The question rises: How do we guarantee this condition? This is the role of the boundary conditions, i.e. when $a_n\le 0$, we need to impose $u=0$. For outflow, i.e. $\partial \Omega _{out}$ we have $a_n>0$ and using this information, we directly obtain

25 $$\begin{aligned} \left\langle u,Du\right\rangle = {\frac{1}{2}}\int _{\partial \Omega _{out}} a_n u^2 {\mathrm {d}{s}}>0, \end{aligned}$$

and we have an energy estimate. We do not discuss well posedness, but we recommend [28, 36] for details regarding that. Now, we transfer our analysis to the discrete framework and imitate this behavior discretely.

#### Discrete Setting

We have to approximate the spatial operator *D* and the boundary condition (B.C), i.e. $Du+B.C$ by an operator of the form ${\underline{\underline{M}}}\,^{-1}( {\underline{\underline{Q}}}\,-{\underline{\underline{\Pi }}}\,) \underline{u}$ where we apply SBP operators as defined in Definition 3.1. The term ${\underline{\underline{M}}}\,^{-1}{\underline{\underline{Q}}}\,\underline{u}$ approximates *Du* and ${\underline{\underline{\Pi }}}\, \underline{u}$ is used to describe *Bu* weakly. Here, the projection operator ${\underline{\underline{\Pi }}}\,$ works only at the boundary points. Note that we must have a ${\underline{\underline{Q}}}\,$ such that ${\underline{\underline{Q}}}\,+{\underline{\underline{Q}}}\,^T$ only contain boundary terms. Looking at the dissipative nature of ${\underline{\underline{M}}}\,^{-1}{\underline{\underline{Q}}}\,\underline{u}$ amounts to study its spectrum. The related eigenvalue problem is

26 $$\begin{aligned} {\underline{\underline{M}}}\,^{-1}({\underline{\underline{Q}}}\,-{\underline{\underline{\Pi }}}\,) \underline{{\tilde{u}}} =\lambda \underline{{\tilde{u}}}. \end{aligned}$$

We denote by $\underline{{\tilde{u}}}^{*,T}$, the adjoint of ${\tilde{u}}$ and multiply (26) with $\underline{{\tilde{u}}}^{*,T}{\underline{\underline{M}}}\,$ to obtain

27 $$\begin{aligned} \underline{{\tilde{u}}}^{*,T}({\underline{\underline{Q}}}\, - {\underline{\underline{\Pi }}}\,) \underline{{\tilde{u}}}= \lambda \underline{{\tilde{u}}}^{*,T}{\underline{\underline{M}}}\,\underline{{\tilde{u}}}= \lambda ||\underline{{\tilde{u}}}||_{{\underline{\underline{M}}}\,}^2. \end{aligned}$$

We transpose (27) and add both equations together. This results in

28 $$\begin{aligned} \underbrace{\underline{{\tilde{u}}}^{*,T}\left( ({\underline{\underline{Q}}}\,+{\underline{\underline{Q}}}\,^T)-({\underline{\underline{\Pi }}}\,+{\underline{\underline{\Pi }}}\,^T) \right) \underline{{\tilde{u}}}}_{:=BT}= 2{\text {Re}}(\lambda )||\underline{{\tilde{u}}}||_{{\underline{\underline{M}}}\,}^2. \end{aligned}$$

The boundary terms (BT) correspond to $\int _{\partial \Omega _{out}} a_n u^2 {\mathrm {d}{s}}$ with a properly chosen ${\underline{\underline{\Pi }}}\,$. Hence, the matrix

29 $$\begin{aligned} ({\underline{\underline{Q}}}\,- {\underline{\underline{\Pi }}}\,)+ ( {\underline{\underline{Q}}}\,-{\underline{\underline{\Pi }}}\, )^T \end{aligned}$$

is positive semi-definite, i.e. the eigenvalues for the spatial operator have a strictly positive real parts only. Note that condition (29) and (21) are the same. Next, we estimate the boundary operators for a linear system such that the conditions in Theorem 3.6 are fulfilled and the pure CG scheme is stable. We start with the continuous energy analysis and derive the estimate above. Afterwards, we translate the result to the discrete FE framework as done for the scalar one-dimensional case, but before, we give the following remark:

##### Remark 4.1

(Periodic Boundary Conditions) As already described in the Introduction 1, periodic boundary conditions for hyperbolic problems together with a pure Galerkin scheme yields stability issues (von Neumann instability). If we consider a Galerkin scheme with the described operators where the used quadrature rule in the numerical integration is the same as the one in the differential operator and periodic boundary conditions in a hyperbolic problem, we have the eigenvalues on the imaginary axis, cf. [27]. Therefore, explicit time-integration schemes of order one or two like Euler method or SSPRK22 lead to instability since they do not include parts of the imaginary axis in their stability regions. In such a case, we have to add stabilization terms (diffusion) to the equation. Further, we want to point out that even with a stable discretization for a linear hyperbolic problem, an unbounded error growth is observed if periodic boundary conditions are imposed [37].

#### Systems of Equations

Next, we will extend our investigation to the general hyperbolic system

30 $$\begin{aligned} \begin{aligned}&\dfrac{\partial U}{\partial t}+A\dfrac{\partial U}{\partial x}+B\dfrac{\partial U}{\partial y}=0,\quad&(x,y)\in \Omega , t>0\\&L_{{\mathbf {n}}}U=G_{{\mathbf {n}}}&(x,y)\in \partial \Omega , t>0 \end{aligned} \end{aligned}$$

where $A,B\in {\mathbb {R}}^{m\times m}$ are the Jacobian matrices of the system, the matrix $L_{{\mathbf {n}}}\in {\mathbb {R}}^{q\times m}$ and the vector $G_{{\mathbf {n}}}\in {\mathbb {R}}^q$ are known, ${{\mathbf {n}}}$ is the local outward unit vector, *q* is the number of boundary conditions to satisfy. We assume that *A*, *B* are constant and that the system (30) is symmetrizable. There exists a symmetric and positive definite matrix *P* such that for any vector ${{\mathbf {n}}}=(n_x,n_y)^T$ the matrix

$$\begin{aligned} C_{{\mathbf {n}}}=A_{{\mathbf {n}}}P \end{aligned}$$

is symmetric with $A_{{\mathbf {n}}}=An_x+Bn_y$. For arbitrary $n_x$, $n_y$ this implies that both *AP* and *BP* are symmetric.

Using the matrix *P*, one can introduce new variables $V=P^{-1}U$. The original variable can be expressed as $U=PV$ and the original system (30) will become

31 $$\begin{aligned} P\dfrac{\partial V}{\partial t}+AP\dfrac{\partial V}{\partial x}+BP \dfrac{\partial V}{\partial y}=0 \Longleftrightarrow \dfrac{\partial U}{\partial t}+AP\dfrac{\partial V}{\partial x}+BP \dfrac{\partial V}{\partial y}=0. \end{aligned}$$

The system (30) admits an energy: if we multiply (30) by $V^T$, we first get

$$\begin{aligned} \begin{aligned} \int _\Omega V^T\dfrac{\partial U}{\partial t}\; {\mathrm {d}{\Omega }}&=-\int _{\Omega } V^T\bigg ( A\dfrac{\partial U}{\partial x}+B\dfrac{\partial U}{\partial y} \bigg )\; {\mathrm {d}{\Omega }}\\&=-\int _\Omega V^T \bigg ( AP \dfrac{\partial V}{\partial x}+BP \dfrac{\partial V}{\partial y}\bigg ) \; {\mathrm {d}{\Omega }}=-\frac{1}{2}\int _{\partial \Omega } V^T C_{{\mathbf {n}}}V \;{\mathrm {d}{\gamma }}\end{aligned} \end{aligned}$$

i.e. setting $E=\frac{1}{2}\int _\Omega V^T U \; {\mathrm {d}{\Omega }}$, we have

$$\begin{aligned} \dfrac{dE}{dt}+\frac{1}{2}\int _{\partial \Omega } V^T C_{{\mathbf {n}}}V \; {\mathrm {d}{\gamma }}=0. \end{aligned}$$

To understand the role of the boundary conditions, we follow what is usually done for conservation laws, we consider the weak form of (30): let $\varphi$ be a regular vector function in space and time. We multiply the equation by $\varphi ^T$, integrate and get:

$$\begin{aligned} \begin{aligned} \int _0^T\int _\Omega \varphi ^T \dfrac{\partial U}{\partial t}\,{\mathrm {d}{\Omega }}\; {\mathrm {d}{t}}&-\int _0^T\int _\Omega \bigg ( \dfrac{\partial \varphi }{\partial x}^T A +\dfrac{\partial \varphi }{\partial y}^T B\bigg ) U\; {\mathrm {d}{\Omega }}\; {\mathrm {d}{t}} \\&+\frac{1}{2}\int _0^T \int _{\partial \Omega } \varphi ^T A_{{\mathbf {n}}}U\; {\mathrm {d}{\gamma }}\; {\mathrm {d}{t}}=0. \end{aligned} \end{aligned}$$

In order to enforce the boundary conditions weakly, we modify this relation by (note that $C_{{\mathbf {n}}}V=A_{{\mathbf {n}}}U$):

32 $$\begin{aligned} \begin{aligned} \int _0^T\int _\Omega \varphi ^T \dfrac{\partial U}{\partial t}\,{\mathrm {d}{\Omega }}\; {\mathrm {d}{t}}&-\int _0^T\int _\Omega \bigg ( \dfrac{\partial \varphi }{\partial x}^T A+\dfrac{\partial \varphi }{\partial y}^T B\bigg ) U\; {\mathrm {d}{\Omega }}\; {\mathrm {d}{t}}\\&\qquad +\frac{1}{2}\int _0^T \int _{\partial \Omega } \varphi ^T A_{{\mathbf {n}}}U\; {\mathrm {d}{\gamma }}\; {\mathrm {d}{t}}\\&\qquad \qquad =\int _0^T\int _{\partial \Omega } \varphi ^T \Pi _{{\mathbf {n}}}\big (L_{{\mathbf {n}}}(U)-G_{{\mathbf {n}}}\big ) \; {\mathrm {d}{\gamma }}\; {\mathrm {d}{t}}. \end{aligned} \end{aligned}$$

The operator $\Pi _{{\mathbf {n}}}$ depends on ${\mathbf {x}}=(x,y)\in \partial \Omega$ and ${{\mathbf {n}}}$ the outward unit normal at ${\mathbf {x}}\in \partial \Omega$. It is chosen in such a way that:

1. For any *t*, the image of the boundary operator $L_{{\mathbf {n}}}(U)$ is the same as the image of $\Pi _{{\mathbf {n}}}L_{{\mathbf {n}}}(U)$ in the weak formulation, i.e. there is no loss of boundary information,
2. If $\varphi =V$, then $\dfrac{{\mathrm {d}{E}}}{ {\mathrm {d}{t}}}< 0$ follows.

A solution to this problem is given by the following: First, let $C_{{\mathbf {n}}}=X_{{\mathbf {n}}}\Lambda _{{\mathbf {n}}}X_{{\mathbf {n}}}^T$ where $\Lambda _{{\mathbf {n}}}$ is a the diagonal matrix containing the eigenvalues of $C_{{\mathbf {n}}}$ and $X_{{\mathbf {n}}}$ is the matrix which rows are the right eigenvectors of $C_{{\mathbf {n}}}$. We have $X_{{\mathbf {n}}}^TX_{{\mathbf {n}}}={\mathbb {I}}$ and choose:

33 $$\begin{aligned} \Pi _{{\mathbf {n}}}\big ( L_{{\mathbf {n}}}(U)-G_{{\mathbf {n}}}\big )= X_{{\mathbf {n}}}\Lambda _{{\mathbf {n}}}^- \begin{pmatrix} R_{{\mathbf {n}}}\\ {\mathbf {0}}_{n-n_0} \end{pmatrix} X_{{\mathbf {n}}}^T- \begin{pmatrix}G_{{\mathbf {n}}}\\ {\mathbf {0}}_{n-n_0}\end{pmatrix}, \end{aligned}$$

where $\Lambda _{{\mathbf {n}}}^-$ are the negative eigenvalues only and *n* denotes the number of unknowns for the system. Here we have introduced the operator $R_{{\mathbf {n}}}$ which is $L_{{\mathbf {n}}}$ written using characteristic variables.

In the following, we explain the implementation steps. To a large content we refer to [28]. To compute $\Pi$ we first consider again the strong implementation of the problem. We have

34 $$\begin{aligned} \frac{{\mathrm {d}{}} }{{\mathrm {d}{t}}}V^TU= -\int _{\partial \Omega } V^T C_{{\mathbf {n}}}V\; {\mathrm {d}{\gamma }}. \end{aligned}$$

Using $C_{{\mathbf {n}}}=X_{{\mathbf {n}}}\Lambda _{{\mathbf {n}}}X_{{\mathbf {n}}}^T$, we obtain

35 $$\begin{aligned} V^T C_{{\mathbf {n}}}V= V^TX_{{\mathbf {n}}}\Lambda _{{\mathbf {n}}}X_{{\mathbf {n}}}^T V= \left( X_{{\mathbf {n}}}^TV \right) ^T \Lambda _{{\mathbf {n}}}\left( X_{{\mathbf {n}}}^TV \right) = \begin{pmatrix} W_{{\mathbf {n}}}^+\\ W_{{\mathbf {n}}}^- \end{pmatrix}^T \begin{pmatrix} \Lambda _{{\mathbf {n}}}^+ &{} 0 \\ 0 &{} \Lambda _{{\mathbf {n}}}^- \end{pmatrix} \begin{pmatrix} W_{{\mathbf {n}}}^+\\ W_{{\mathbf {n}}}^- \end{pmatrix} \end{aligned}$$

with $W_{{\mathbf {n}}}^+=\left( X_{{\mathbf {n}}}^TV \right) ^+$ are the ingoing waves and they have the size of the positive eigenvalues $\Lambda _{{\mathbf {n}}}^+$. Analogously, $W_{{\mathbf {n}}}^-=\left( X_{{\mathbf {n}}}^TV \right) ^-$ are the outgoing waves with size of $\Lambda _{{\mathbf {n}}}^-$. A general homogeneous boundary condition is ${W_{{\mathbf {n}}}^-=R_{{\mathbf {n}}}W_{{\mathbf {n}}}^+}$, since with that, and a proper choice of $R_{{\mathbf {n}}}$, we get

36 $$\begin{aligned} V^T C_{{\mathbf {n}}}V= (W_{{\mathbf {n}}}^+)^T \left( \Lambda _{{\mathbf {n}}}^++R_{{\mathbf {n}}}^T\Lambda _{{\mathbf {n}}}^-R_{{\mathbf {n}}}\right) W_{{\mathbf {n}}}^+\ge 0 \end{aligned}$$

and so the decrease of energy in (34) if the matrix in the bracket is positive semidefinite.

Next, we will impose the boundary conditions weakly. Assume now that we have chosen an $R_{{\mathbf {n}}}$ such that

37 $$\begin{aligned} \left( \Lambda _{{\mathbf {n}}}^++R_{{\mathbf {n}}}^T\Lambda _{{\mathbf {n}}}^-R_{{\mathbf {n}}}\right) \ge 0. \end{aligned}$$

Here, the existence of such $R_{{\mathbf {n}}}$ is ensured through our assumption that our boundary value problem (30) is well posed, c.f. [28]. The energy is given

38 $$\begin{aligned} \int _{\Omega } V^T \dfrac{\partial U}{\partial t}\; {\mathrm {d}{\Omega }}+ \frac{1}{2}\int _{\partial \Omega } V^TA_{{\mathbf {n}}}U {\mathrm {d}{\gamma }}=\int _{\partial \Omega }V^T \Pi _{{\mathbf {n}}}\left( W_{{\mathbf {n}}}^--R_{{\mathbf {n}}}W_{{\mathbf {n}}}^+ \right) \; {\mathrm {d}{\gamma }}. \end{aligned}$$

We add the transpose of (38) to itself and consider

39 $$\begin{aligned} \begin{aligned} \frac{{\mathrm {d}{}} }{{\mathrm {d}{t}}}\int _{\partial \Omega } V^TU\; {\mathrm {d}{\Omega }}=&-\int _{\partial \Omega }V^TA_{{\mathbf {n}}}U{\mathrm {d}{\gamma }}\\&+\int _{\partial \Omega } V^T \Pi _{{\mathbf {n}}}\left( W_{{\mathbf {n}}}^--R_{{\mathbf {n}}}W_{{\mathbf {n}}}^+ \right) + \left( W_{{\mathbf {n}}}^--R_{{\mathbf {n}}}W_{{\mathbf {n}}}^+ \right) ^T\Pi _{{\mathbf {n}}}^TV \; {\mathrm {d}{\gamma }}. \end{aligned} \end{aligned}$$

We define $\tilde{\Pi }_{{\mathbf {n}}}$ such that $V^T \Pi _{{\mathbf {n}}}=(W_{{\mathbf {n}}}^-)^T\tilde{\Pi }_{{\mathbf {n}}}$ and get for the integrands

40 $$\begin{aligned} \begin{aligned}&-(W_{{\mathbf {n}}}^+)^T\Lambda _{{\mathbf {n}}}^+W_{{\mathbf {n}}}^+-(W_{{\mathbf {n}}}^-)^T\Lambda _{{\mathbf {n}}}^-W_{{\mathbf {n}}}^- +(W_{{\mathbf {n}}}^-)^T\tilde{\Pi }_{{\mathbf {n}}}\left( W_{{\mathbf {n}}}^--R_{{\mathbf {n}}}W_{{\mathbf {n}}}^+ \right) \\&\quad \quad + \left( W_{{\mathbf {n}}}^--R_{{\mathbf {n}}}W_{{\mathbf {n}}}^+ \right) ^T\tilde{\Pi }_{{\mathbf {n}}}^T W_{{\mathbf {n}}}^-. \end{aligned} \end{aligned}$$

Collecting the terms, we obtain

41 $$\begin{aligned} \begin{pmatrix} W_{{\mathbf {n}}}^+\\ W_{{\mathbf {n}}}^- \end{pmatrix}^T \underbrace{\begin{pmatrix} -\Lambda _{{\mathbf {n}}}^+ &{} - R_{{\mathbf {n}}}^T\tilde{\Pi }_{{\mathbf {n}}}^T \\ - \tilde{\Pi }_{{\mathbf {n}}}R_{{\mathbf {n}}}&{} - \Lambda _{{\mathbf {n}}}^- +\tilde{\Pi }_{{\mathbf {n}}}+\tilde{\Pi }_{{\mathbf {n}}}^T \end{pmatrix}}_{=:WB} \begin{pmatrix} W_{{\mathbf {n}}}^+\\ W_{{\mathbf {n}}}^- \end{pmatrix}. \end{aligned}$$

We must select $\tilde{\Pi }_{{\mathbf {n}}}$ such that the matrix *WB* is negative definite. Now, let us use the strong condition (37). By adding and subtracting, we obtain

42 $$\begin{aligned} \underbrace{\begin{pmatrix} W_{{\mathbf {n}}}^+\\ W_{{\mathbf {n}}}^- \end{pmatrix}^T \begin{pmatrix} R^T_{{\mathbf {n}}}\Lambda _{{\mathbf {n}}}^- R_{{\mathbf {n}}}&{} - R_{{\mathbf {n}}}^T\tilde{\Pi }_{{\mathbf {n}}}^T \\ -\tilde{\Pi }_{{\mathbf {n}}}R_{{\mathbf {n}}}&{}- \Lambda _{{\mathbf {n}}}^- +\tilde{\Pi }_{{\mathbf {n}}}+\tilde{\Pi }_{{\mathbf {n}}}^T \end{pmatrix} \begin{pmatrix} W_{{\mathbf {n}}}^+\\ W_{{\mathbf {n}}}^- \end{pmatrix}}_{=:Q_w} \underbrace{-\left( (W_{{\mathbf {n}}}^+)^T(\Lambda _{{\mathbf {n}}}^++R_{{\mathbf {n}}}^T\Lambda _{{\mathbf {n}}}^-R_{{\mathbf {n}}}) W_{{\mathbf {n}}}^+\right) .}_{\le 0 \text { by }(37).} \end{aligned}$$

By rearranging and choosing $\tilde{\Pi }_{{\mathbf {n}}}=\Lambda _{{\mathbf {n}}}^-$, we get

$$\begin{aligned} Q_w= \begin{pmatrix} R_{{\mathbf {n}}}W_{{\mathbf {n}}}^+\\ W_{{\mathbf {n}}}^- \end{pmatrix}^T \underbrace{ \begin{pmatrix} 1 &{} -1 \\ -1&{}1 \end{pmatrix}}_{=:G_{{\mathbf {n}}}} \otimes \Lambda ^- \begin{pmatrix} R_{{\mathbf {n}}}W_{{\mathbf {n}}}^+\\ W_{{\mathbf {n}}}^- \end{pmatrix}. \end{aligned}$$

Since $G_{{\mathbf {n}}}$ has the eigenvalues 0 and 2 and we obtain stability

43 $$\begin{aligned} \dfrac{d}{dt} \frac{1}{2} \int _\Omega V^TU\; {\mathrm {d}{x}} +\frac{1}{2}\int _{\partial \Omega } V^TA_{{\mathbf {n}}}U\; {\mathrm {d}{\gamma }}\le \frac{1}{2} \int _{\partial \Omega }V^T\Pi _{{\mathbf {n}}}\big ( L_{{\mathbf {n}}}(U)-G_{{\mathbf {n}}}\big )\; {\mathrm {d}{\gamma }}\end{aligned}$$

thanks to (42) and (36). We will give a concrete example in Sect. 5.3.

## Numerical Simulations

Here, we demonstrate that a pure Galerkin scheme is stable if we impose the boundary conditions weakly and use an adequate boundary operator as described in Sect. 4. We consider several different examples and analyze different properties in this context (error behavior, eigenvalues, etc.). As basis functions, we use Bernstein or Lagrange polynomials of different orders resulting in Galerkin schemes of second to fourth order on triangular meshes. We denote with $B1,\;B2,\;B3$ the Galerkin method using Bernstein polynomials with polynomial order 1, 2 or 3 similar denoting $P1,\;P2,\;P3$ by applying a Lagrange basis. The basic implementation is done in the RD framework, see [38]. The two approaches only differ slightly. The time integration is done via strong stability preserving Runge-Kutta methods of second to fourth order, see [39] for details. We use always the same order for space and time discretization.

### Two-Dimensional Scalar Equations

We consider a two-dimensional scalar hyperbolic equation of the form

44 $$\begin{aligned} \dfrac{\partial U}{\partial t} + {\mathbf {a}}(x,y)\cdot \nabla U = 0, \quad (x,y) \in \Omega , \ t > 0, \end{aligned}$$

where ${\mathbf {a}}= (a,b)$ is the advection speed and $\Omega$ the domain. In this subsection, the initial condition is given by

$$\begin{aligned} U(x,y,0)={\left\{ \begin{array}{ll}{ll} \mathrm {e^{-40r^2}},&{}\quad \text { if } r=\sqrt{(x-x_0)^2{+}(y-y_0)^2}<0.25 \\ 0,&{}\quad \text { otherwise }. \end{array}\right. }\\ \end{aligned}$$

It is a small bump with height one located at $(x_0,y_0)$. We consider homogeneous boundary conditions $G_{{\mathbf {n}}}\equiv 0$ and further let the boundary matrix $L_{{\mathbf {n}}}$ be the identity matrix at the inflow part of $\partial \Omega$ (i.e. $\partial \Omega ^-)$. The boundary conditions reads $L_{{\mathbf {n}}}U=U=0$ for $(x,y)\in \partial \Omega ^-, \;t >0$ which means that the incoming waves are set to zero.

#### Linear Advection

**Fig. 1:**
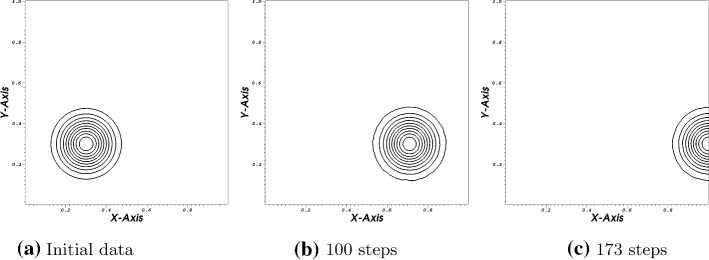
4-th order scheme in space and time

In our first test, we are considering the linear advection equation in $\Omega =[0,1]^2$. The advection speed is assumed to be constant. The components of the speed vector ${\mathbf {a}}$ are given by $(a,b)^T=(1,0)$ and so the flux is given by ${{\mathbf {f}}}(U)=\varvec{a}U$ with $\varvec{a}=(1,0)$. We have inflow / outflow conditions on the left / right boundaries and periodic boundary condition on the horizontal boundaries. In our first test, we use Bernstein polynomials and a fourth order C.G. scheme. The boundary operators are computed using the technique developed in Sect. 4 where the positive eigenvalues are set to zero and the negative ones are used in the construction of $\Pi$. For the time discretization we apply strong stability preserving Runge–Kutta (SSPRK) scheme with 5 stages and fourth order with CFL $=$ 0.3. We use 1048 triangles. In Fig. (), we plot the results at three times. Clearly, the scheme is stable, also at the outflow boundary. The maximum value is at the end 1.001 and the minimum is $-\,0.0199$ where the starting values are 1.000 and 0.000 (Fig. 1).

Next, we check the real parts of the eigenvalues of our problem using formula (21) for different orders, different bases (Bernstein and Lagrange) and different meshes. For the calculation of the eigenvalues of (21), we use a Petsc routine [40, 41] which can calculate up to 500 eigenvalues.^4^ Have in mind that in contrast to DG and multi-block FD setting, the mass matrix in the pure Galerkin scheme is not block diagonal. Therefore, we can not split the eigenvalue calculation to each block matrix and have to consider the whole matrix ${\underline{\underline{M}}}\,$ and therefore ${\underline{\underline{Q}}}\,$. Different from before, we consider the complete skew-symmetric spatial operator ${\underline{\underline{Q}}}\,+{\underline{\underline{Q}}}\,^T$ in the whole domain and not in one element only, where it is equal to ${\underline{\underline{B}}}\,$. Every coefficient of the numerical approximation belongs to one degree of freedom and we obtain the same number of eigenvalues as number of DoFs are used. However, for the coefficients which belong to DoFs inside the domain, in all calculations we obtain zero up to machine precision. To get useful results, we decrease the number of elements in the following calculations and provide only the most negative and positive ones in Tables 1, 2 and 3 where we give results with and without the application of the SAT boundary operators.

**Table 1:** Eigenvalue of the operators (29) with and without the boundary operators using *P*1/*B*1 (41DoFs)

neg. eigen. of ${\underline{\underline{Q}}}\,+{\underline{\underline{Q}}}\,^T$	pos. eigen. of ${\underline{\underline{Q}}}\,+{\underline{\underline{Q}}}\,^T$	neg. eig. for BT from (29)	pos. eig. for BT from (29)
$-\,0.2317$	0.2317	$-\,0.3135$	$6.0289 \; \cdot 10^{-17}$
$-\,0.1839$	0.1839	$-\,0.2555$	$3.8787\; \cdot 10^{-17}$
$-\,0.1250$	0.1250	$-\,0.2317$	$3.0097\; \cdot 10^{-17}$
$-\,6.6074\; \cdot 10^{-2}$	$6.6074\; \cdot 10^{-2}$	$-\,0.1848$	$2.3845\; \cdot 10^{-17}$
$-\,5.9935\; \cdot 10^{-2}$	$5.9935\; \cdot 10^{-2}$	$-\,0.1839$	$1.7762\; \cdot 10^{-17}$
$-\,7.2852\; \cdot 10^{-17}$	$3.9582\; \cdot 10^{-17}$	$-\,0.1250$	$1.2997\; \cdot 10^{-17}$
$-\,4.0170\; \cdot 10^{-17}$	$3.4527\; \cdot 10^{-17}$	$-\,0.1181$	$9.7095\; \cdot 10^{-18}$
$-\,3.0744\; \cdot 10^{-17}$	$2.6742\; \cdot 10^{-17}$	$-\,7.7263\; \cdot 10^{-2}$	$9.4396\; \cdot 10^{-18}$
$-\,2.9953\; \cdot 10^{-17}$	$2.4023\; \cdot 10^{-17}$	$-\,6.6074\; \cdot 10^{-2}$	$9.4396\; \cdot 10^{-18}$
$-\,2.3732\; \cdot 10^{-17}$	$1.8552\; \cdot 10^{-17}$	$-\,5.9935\; \cdot 10^{-2}$	$6.6812\; \cdot 10^{-18}$
$-\,1.9299\; \cdot 10^{-17}$	$1.2938\; \cdot 10^{-17}$	$-\,7.2894\; \cdot 10^{-17}$	$5.6739\; \cdot 10^{-18}$

**Table 2:** Eigenvalue of the operators (29) with and without the boundary operators using *B*2 (145DoFs)

neg. eigen. of ${\underline{\underline{Q}}}\,+{\underline{\underline{Q}}}\,^T$	pos. eigen. of ${\underline{\underline{Q}}}\,+{\underline{\underline{Q}}}\,^T$	neg. eig. for BT from (29)	pos. eig. for BT from (29)
$-\,0.1343$	0.1343	$-\,0.1924$	$2.8085\; \cdot 10^{-16}$
$-\,0.1186$	0.1187	$-\,0.1746$	$2.5390\; \cdot 10^{-16}$
$-\,9.7804\; \cdot 10^{-2}$	$9.7804\; \cdot 10^{-2}$	$-\,0.1524$	$2.4456\; \cdot 10^{-16}$
$-\,6.1147\; \cdot 10^{-2}$	$6.1147\; \cdot 10^{-2}$	$-\,0.1343$	$2.2495\; \cdot 10^{-16}$
$-\,5.8452\; \cdot 10^{-2}$	$5.8451\; \cdot 10^{-2}$	$-\,0.1186$	$2.2016\; \cdot 10^{-16}$
$-\,2.6008\; \cdot 10^{-2}$	$2.6008\; \cdot 10^{-2}$	$-\,0.1088$	$2.0043\; \cdot 10^{-16}$
$-\,1.6694\; \cdot 10^{-2}$	$1.6694\; \cdot 10^{-2}$	$-\,9.7804\; \cdot 10^{-2}$	$1.9144\; \cdot 10^{-16}$
$-\,1.0862\; \cdot 10^{-2}$	$1.0862\; \cdot 10^{-2}$	$-\,6.9258\; \cdot 10^{-2}$	$1.8695\; \cdot 10^{-16}$
$-\,9.4054\; \cdot 10^{-3}$	$9.4054\; \cdot 10^{-3}$	$-\,6.1147\; \cdot 10^{-2}$	$1.8192\; \cdot 10^{-16}$
$-\,2.7617\; \cdot 10^{-16}$	$2.8085\; \cdot 10^{-16}$	$-\,5.8452\; \cdot 10^{-2}$	$1.7825\; \cdot 10^{-16}$
$-\,2.5757\; \cdot 10^{-16}$	$2.5390\; \cdot 10^{-16}$	$-\,3.0333\; \cdot 10^{-2}$	$1.7762\; \cdot 10^{-16}$

**Table 3:** Eigenvalue of the operators (29) with and without the boundary operators using *B*3 (313DoFs)

neg. eigen. of ${\underline{\underline{Q}}}\,+{\underline{\underline{Q}}}\,^T$	pos. eigen. of ${\underline{\underline{Q}}}\,+{\underline{\underline{Q}}}\,^T$	neg. eig. for BT from (29)	pos. eig. for BT from (29)
$-\,9.3746\; \cdot 10^{-2}$	$9.3746\; \cdot 10^{-2}$	$-\,0.1417$	$2.2109\; \cdot 10^{-16}$
$-\,8.6774\; \cdot 10^{-2}$	$8.6774\; \cdot 10^{-2}$	$-\,0.1345$	$2.1270\; \cdot 10^{-16}$
$-\,7.7346\; \cdot 10^{-2}$	$7.7346\; \cdot 10^{-2}$	$-\,0.1260$	$2.0375\; \cdot 10^{-16}$
$-\,5.1492\; \cdot 10^{-2}$	$5.1492\; \cdot 10^{-2}$	$-\,9.3746\; \cdot 10^{-2}$	$1.9449\; \cdot 10^{-16}$
$-\,5.0243\; \cdot 10^{-2}$	$5.0243\; \cdot 10^{-2}$	$-\,9.1241\; \cdot 10^{-2}$	$1.9060\; \cdot 10^{-16}$
$-\,3.1541\; \cdot 10^{-2}$	$3.1541\; \cdot 10^{-2}$	$-\,8.6774\; \cdot 10^{-2}$	$1.8710\; \cdot 10^{-16}$
$-\,2.3206\; \cdot 10^{-2}$	$2.3206\; \cdot 10^{-2}$	$-\,7.7354\; \cdot 10^{-2}$	$1.8543\; \cdot 10^{-16}$
$-\,1.6530\; \cdot 10^{-2}$	$1.6530\; \cdot 10^{-2}$	$-\,5.7862\; \cdot 10^{-2}$	$1.7657\; \cdot 10^{-16}$
$-\,1.4887\; \cdot 10^{-2}$	$1.4887\; \cdot 10^{-2}$	$-\,5.1492\; \cdot 10^{-2}$	$1.6555\; \cdot 10^{-16}$
$-\,4.4006\; \cdot 10^{-3 }$	$4.4006\; \cdot 10^{-3}$	$-\,5.0243\; \cdot 10^{-2}$	$1.6338\; \cdot 10^{-16}$
$-\,3.0050\; \cdot 10^{-3}$	$3.0050\; \cdot 10^{-3}$	$-\,3.6266\; \cdot 10^{-2}$	$1.6222\; \cdot 10^{-16}$
$-\,2.1187\; \cdot 10^{-3}$	$2.1187\; \cdot 10^{-3}$	$-\,3.1541\; \cdot 10^{-2}$	$1.6088\; \cdot 10^{-16}$
$-\,1.8526\; \cdot 10^{-3}$	$1.8526\; \cdot 10^{-3}$	$-\,2.8790\; \cdot 10^{-2}$	$1.5858\; \cdot 10^{-16}$
$-\,2.1399\; \cdot 10^{-16}$	$2.2109\; \cdot 10^{-16}$	$v2.3210\; \cdot 10^{-2}$	$1.5731\; \cdot 10^{-16}$

We see from Tables 1, 2 and 3 that the the boundary operator decreases the negative eigenvalues and forces the positive ones to zero (up to machine precision). For third and fourth order, we print only the case using Bernstein polynomials. The applications of Lagrange polynomials lead only to slightly bigger amounts of positive and negative eigenvalues of the ${\underline{\underline{Q}}}\,+{\underline{\underline{Q}}}\,^T$ operator (i.e maximum eigenvalue is 0.11713334374388217 for *P*3). However, the results are similar after applying the SAT procedure, we obtain only negative or zero eigenvalues.

We also mention that for higher degrees and more DoFs, we may strengthen the SAT terms to guarantee that the eigenvalues are negative and /or forced to zero. All of our investigations are in accordance with the analysis done in Sect. 4.1 and all of our calculations demonstrate that a pure Galerkin scheme is stable if a proper boundary procedure is used.

##### Remark 5.1

Finally, we did a couple of additional simulations changing both, the domain $\Omega$ (circles, pentagons, etc.) and the speed vector including also some horizontal movement. All of our calculations remained stable if the boundary approach from Sect. 4 was used.

This is in contradiction of a common belief in the hyperbolic research community about continuous Galerkin schemes.

But what are the reasons for this belief? In our opinion, one of the major issues is that the chosen quadrature rule in the numerical integration differs from the the one used in the differential operators and without artificial stabilization terms the continuous Galerkin scheme collapses, and the corresponding ${\underline{\underline{Q}}}\,$ matrix does not become almost skew-symmetric.

#### Inexactness of the Quadrature Rule

**Fig. 2:**
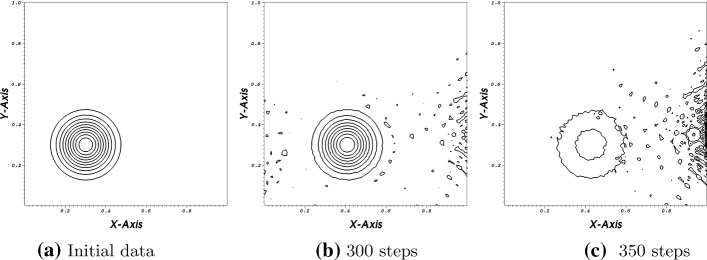
4-th order scheme in space and time

To support our statement, we provide the following example. We consider the same problem as before, but in the Galerkin scheme we lower the accuracy of our quadrature rule to calculate the mass matrix and the conditions at the boundary procedure. Before, we used always a quadrature rule which is accurate up to sixth order. Then, we lower the quadrature rule for the surface integral to five, the rest remain the same. Please be aware that in the Galerkin approach, we apply integration by parts before formulating the variation formulation. We decrease the CFL number to 0.01 for stability reasons. However, as it is shown in Fig. 2 the scheme crashes after some time even with this super low CFL number. In Fig. 2c, the structure of the bump can still be seen, but, simultaneously, the minimum value is $\approx -\,2.996$ and the maximum value is around 2.7 (Fig. 2).

Additional time steps will lead to a complete crash of the test. At 400 steps the maximum value is 74.45 and the minimum is at $-\,82.15$. Here, again nothing has changed from the calculations before except that the quadrature rules are changed which leads to an error in the interior of the spatial matrix ${\underline{\underline{Q}}}\,$, which cannot be stabilized with the SAT boundary treatment. We will focus on this test again in the second part of the paper series [42] to demonstrate the entropy correction term as presented in [38] and applied in [43, 44] can also be seen as a stabilization factor for linear problems.

#### Linear Rotation

In the next test we consider an advection problem with variable coefficients. The speed vector has components

$$\begin{aligned} a = 2\pi y, \ b = -\,2\pi x. \end{aligned}$$

The initial and boundary conditions are given by

$$\begin{aligned}&U(x,y,0)={\left\{ \begin{array}{ll}{ll} \mathrm {e^{-40r^2}}, &{}\quad \text { if } r=\sqrt{x^2+(y-0.5)^2}<0.25, \\ 0, &{}\quad \text { otherwise } \end{array}\right. }\\&U=0, \quad (x,y)\in \partial \Omega , \; t>0. \end{aligned}$$

**Fig. 3:**
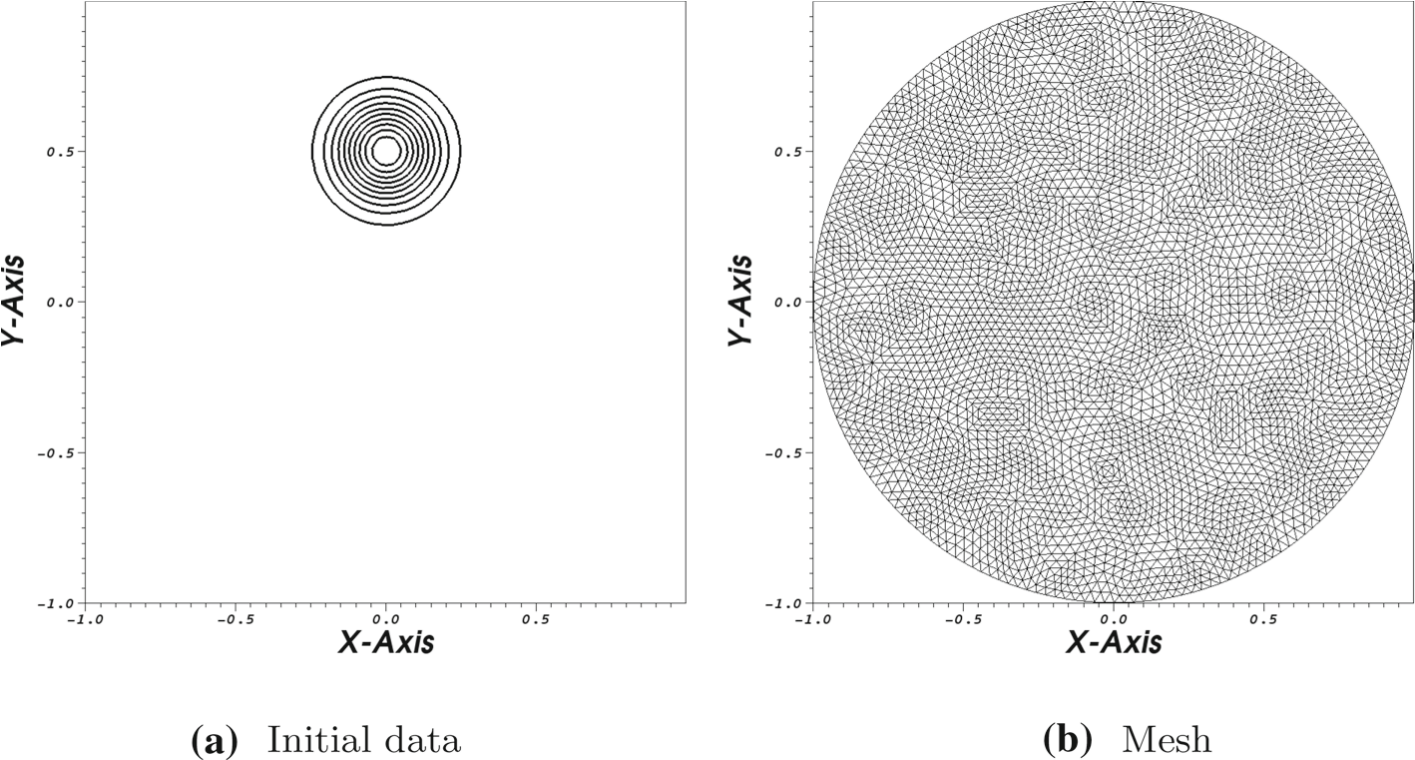
4-th order scheme in space and time

The problem is defined on the unit disk ${\mathbb {D}}=\{(x,y)\in {\mathbb {R}}^2| \sqrt{x^2+y^2}<1 \}$. The small bump rotates in the clockwise direction in a circle around zero. In Fig. 3a the initial state is presented where Fig. 3b shows the used mesh (Fig. 3).

**Fig. 4:**
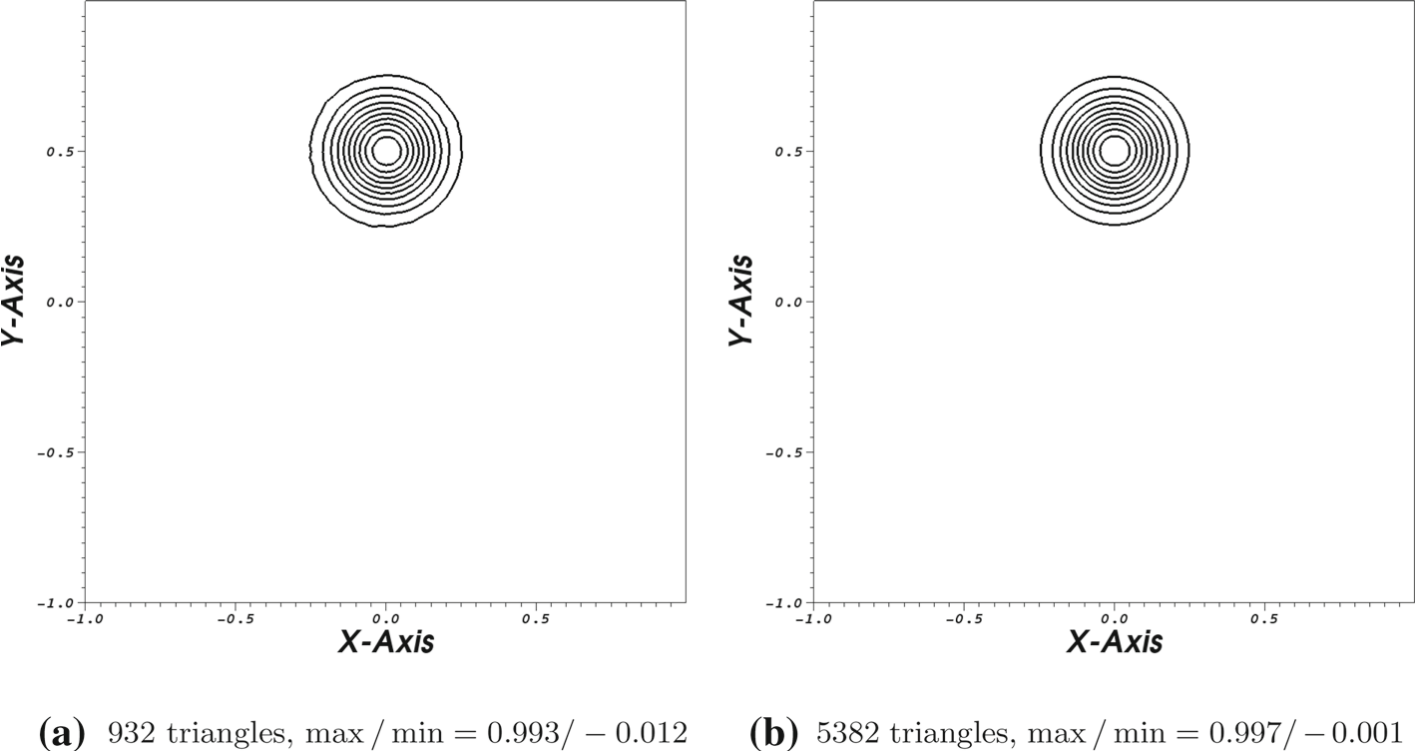
4-th order scheme in space and time

We apply an unstructured triangular mesh with 932 triangles. In the second calculation 5382 triangles are used. The time integration is again done via a SSPRK54 scheme with CFL=0.2. A pure continuous Galerkin scheme with Bernstein polynomials is used for the space discretization. Due to the variable coefficient problem, we apply the splitting technique as described in [27, 31] and see 3.7. The volume term is split into a symmetric and anti-symmetric part, see for details the mentioned literature. The boundary operator is estimated via the approach presented in 4.1.2. In Fig. 4, the results are presented after two rotations of the bump. Using 932 triangles, we obtain a maximum value of 0.993 and a minimum value fo $-\,0.012$. Increasing the number of triangles, the maximum value after two rotations is 9.997 where the minimum value is $-\,0.001$. This test again verifies that our scheme remains stable only through our boundary procedure (Fig. 4).

**Fig. 5:**
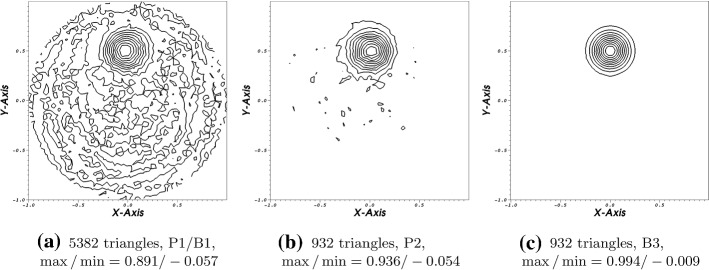
2,3,4-th order scheme in space and time

We compute this problem up to ten rotations for different orders. We observe that all of our calculation remain stable both using Lagrange or Bernstein polynomials as can be seen for example in Fig. 5. In the captions, we mentioned the respective maximum and minimum values and applied 10 contour lines to to divide the different value regions. Especially, in Fig. 5a one can imagine some stability issues. However, this is not the case. Here, the calculations demonstrated some numerical inaccuracies, but the calculation remains stable as can be read of the absolute maximum and minimum values. We recognize also that compared to the others the hight of the bump is decreasing. This behavior suggest a certain amount of artificial dissipation. We obtain the most accurate results using the fourth order scheme which is not surprising (Fig. 5).

**Fig. 6:**
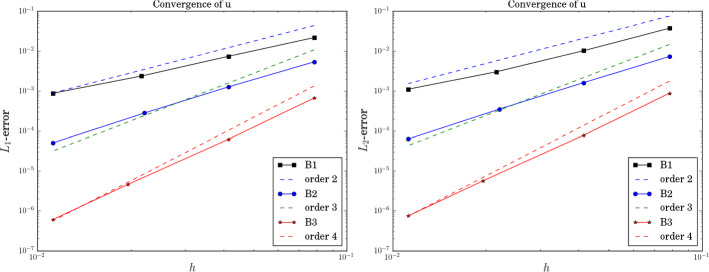
$t=1$, $L_1$-error and $L_2$-error

Finally, we analyze the error behavior and calculate the order. We use again the SSPRK schemes of the same order (Fig. 6).

We recognize a slight decrease of the order similar to the observation made in [45] which was up to our knowledge the first ones who described it. The investigation of the decreased order of accuracy is not the main focus of this paper, where we focus on the stability properties of the pure continuous Galerkin scheme.

### One-Dimensional Wave Equation

As a first example for systems with non-homogeneous boundary condition, we consider the linear wave equation

$$\begin{aligned} \dfrac{\partial ^2 u}{\partial t^2}-\dfrac{\partial ^2 u}{\partial t^2} = 0 \quad t>0, \quad x\in (0,1), \end{aligned}$$

By applying a change of variables ${\tilde{u}}:= \partial _x u$ and ${\tilde{v}}=-\,\partial _t u$, the wave equation can be rewritten as a first order hyperbolic system of conservation laws

45 $$\begin{aligned} \begin{aligned} \dfrac{\partial {\tilde{u}}}{\partial t}+ \dfrac{\partial {\tilde{v}}}{\partial x}&=0,\\ \dfrac{\partial {\tilde{v}}}{\partial t}+\dfrac{\partial {\tilde{u}}}{\partial x}&=0, \end{aligned} \end{aligned}$$

which is sometimes referred to as the one-dimensional acoustic problem. The system (45) can also be expressed through the linear system

46 $$\begin{aligned} \dfrac{\partial U}{\partial t}+ A \dfrac{\partial U}{\partial x}=0 \text { with } U=\begin{pmatrix} {\tilde{u}}\\ {\tilde{v}} \end{pmatrix} \text {and coefficient matrix } A= \begin{pmatrix} 0 &{} 1\\ 1 &{} 0 \end{pmatrix}. \end{aligned}$$

which we consider in the following. We assume the sinusoidal boundary conditions:

$$\begin{aligned}&x=0: \dfrac{1}{\sqrt{2}}\begin{pmatrix} 1 &{} 1 \\ 1 &{}-\,1 \end{pmatrix}\begin{pmatrix} {\tilde{u}}\\ {\tilde{v}} \end{pmatrix} =\begin{pmatrix} \sin t \\ 0\end{pmatrix}, \\&x=1: \dfrac{1}{\sqrt{2}}\begin{pmatrix} 1 &{} 1 \\ 1 &{}-\,1 \end{pmatrix}\begin{pmatrix} {\tilde{u}}\\ {\tilde{v}} \end{pmatrix} =\begin{pmatrix} 0 \\ \sin t \end{pmatrix}. \end{aligned}$$

**Fig. 7:**
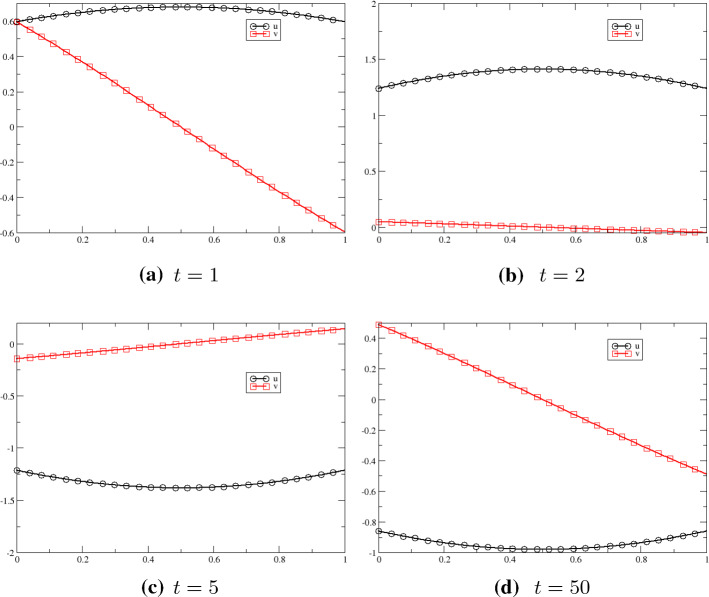
Results for the wave problem (45) and $t=1,2,5,50$, 3rd order scheme in space and time. We have 100 cells (199 degrees of freedom), $\hbox {CFL}=0.1$

To determine the boundary operators, we calculate the eigenvalues and the eigenvectors of *A* following the ideas of Sect. 4.1.2. We obtain the eigenvalues $\lambda _{1/2}= \pm 1$ and

$$\begin{aligned} X=\dfrac{1}{\sqrt{2}}\begin{pmatrix} 1 &{} 1 \\ 1 &{}-\,1 \end{pmatrix}=X^T , \end{aligned}$$

where the rows are the eigenvectors. It is $X^TX={\mathbb {I}}$. We assume that (the subscripts “0” and “1” refers to the end points of [0, 1])

$$\begin{aligned} \Pi _0\big ( M_0(U)-g_0\big )&=\begin{pmatrix} -R_0 &{} 1\\ 0&{}0\end{pmatrix}X^TU-\begin{pmatrix}\sin t \\ 0\end{pmatrix}\\ \Pi _1\big ( M_1(U)-g_1\big )&=\begin{pmatrix} 0&{}0\\ 1&{}-R_1\end{pmatrix}X^TU-\begin{pmatrix}0 \\ \sin t \end{pmatrix} \end{aligned}$$

with $|R_0|,\;|R_1|<1$. Described in [28], the problem is well posed in $L^2([0,1])$. For the time integration, we apply the SSPRK method of third order given in [39] and the space discretization is done via a pure Galerkin scheme of third order using Lagrange polynomials. The CFL condition is set to 0.4. For 100 cells and a regular mesh, we have the results displayed in Fig. 7. We tested it up to $t=50$ without any stability problems. The Galerkin scheme gives us numerical approximations in a way as expected and determined from the theory (Fig. 7).

**Fig. 8:**
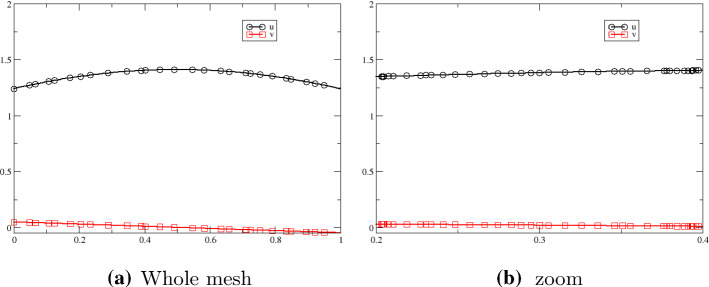
Results for the problem and $t=2$, irregular mesh, 3rd order scheme in space and time. We have 100 cells (199 degrees of freedom), $\hbox {CFL}=0.1$

Under the same terms and conditions, we ran the test again now with a random mesh. Figure 8 demonstrates the results at $t=2$ with a zoom in in Fig. 8b to highlight the mesh points. Indeed, no visible difference can be seen between Figs. 7b and 8a (Fig. 8).

### R13 Sub-model for Heat Conduction

In our last simulation, we consider the steady R13 sub-model for heat conduction investigated in [46, 47]. It reads

47 $$\begin{aligned} \begin{aligned}&\text {div } s =f,\\&\text { grad }\theta +\text { div }{\mathbf {R}}=-\,\frac{s}{\tau },\\&\frac{1}{2}\big (\text { grad} s +(\text {grad }s)^T)=-\,\frac{{\mathbf {R}}}{\tau }, \end{aligned} \end{aligned}$$

in $\Omega =\{(x,y)|\frac{1}{2}\le \sqrt{x^2+y^2}\le 1\}\in {\mathbb {R}}^2$. The outer boundary will be denoted by $\Gamma _1$ and the inner circle is $\Gamma _0$. The process includes a scalar temperature $\theta \in {\mathbb {R}}$, a vector values heat flux $s\in {\mathbb {R}}^2$, and a symmetric tensorial variable ${\mathbf {R}}$ represented by a symmetric $2\times 2$ matrix. $\tau$ is a constant relaxation time.

We set

$$\begin{aligned} s=(s_x,s_y), {\mathbf {R}}=\begin{pmatrix} R_{xx} &{} R_{xy}\\ R_{xy} &{} R_{yy}\end{pmatrix} \end{aligned}$$

If $U=(\theta , s, {\mathbf {R}})$ with ${\mathbf {R}}=(R_{xx},R_{xy},R_{yy})$ the system (47) can be rewritten as:

$$\begin{aligned} A\dfrac{\partial U}{\partial x}+B \dfrac{\partial U}{\partial y}=0. \end{aligned}$$

In the following applications, we will consider the unsteady version of (47)

$$\begin{aligned} \dfrac{\partial U}{\partial t}+A\dfrac{\partial U}{\partial x}+B \dfrac{\partial U}{\partial y}=0 \end{aligned}$$

with boundary conditions that will be detailed in the next part of this section. The aim is to look for a steady solution of this system, and hence to develop a time marching approach. With $\alpha \in {\mathbb {R}}$, the matrix $\cos \alpha A+\sin \alpha B$ reads

48 $$\begin{aligned} A_\alpha =\begin{pmatrix} 0&{}\cos \alpha &{} \sin \alpha &{} 0 &{} 0 &{} 0 &{}\\ \cos \alpha &{} 0 &{} 0 &{} \cos \alpha &{} \sin \alpha &{} 0\\ \sin \alpha &{} 0 &{} 0 &{} 0 &{} \cos \alpha &{} \sin \alpha \\ 0 &{} \cos \alpha &{} 0 &{}0 &{} 0 &{}0\\ 0&{} \frac{\sin \alpha }{2} &{}\frac{\cos \alpha }{2}&{} 0&{} 0&{} 0\\ 0&{}0&{}\sin \alpha &{} 0&{}0&{}0 \end{pmatrix} \end{aligned}$$

and we notice that the system (47) is symmetrizable. The symmetrizer is $P=\mathop {{\mathrm {diag}}}(1,1,1,1,\frac{1}{2},1)$ and together, we obtain

$$\begin{aligned} A_\alpha P=\begin{pmatrix} 0&{}\cos \alpha &{} \sin \alpha &{} 0 &{} 0 &{} 0 &{}\\ \cos \alpha &{} 0 &{} 0 &{} \cos \alpha &{} \frac{\sin \alpha }{2} &{} 0\\ \sin \alpha &{} 0 &{} 0 &{} 0 &{} \frac{\cos \alpha }{2} &{} \sin \alpha \\ 0 &{} \cos \alpha &{} 0 &{}0 &{} 0 &{}0\\ 0&{} \frac{{\sin \alpha }}{2} &{}\frac{{\cos \alpha }}{2}&{} 0&{} 0&{} 0\\ 0&{}0&{}\sin \alpha &{} 0&{}0&{}0 \end{pmatrix}=B_\alpha . \end{aligned}$$

$B_\alpha$ is symmetric and to estimate the boundary operator, we need the eigenvalues and eigenvectors of $A_{{\mathbf {n}}}$. The eigenvectors are

$$\begin{aligned} \begin{aligned} R= \begin{pmatrix} 1 &{} 1 &{} 0 &{} 0 &{} -\,1 &{}-\,\cos ^2\alpha \\ \sqrt{2}\cos \alpha &{} -\,\sqrt{2}\cos \alpha &{} -\,\frac{\sqrt{2}}{2}\sin \alpha &{}\frac{\sqrt{2}}{2}\sin \alpha &{} 0&{}0\\ \sqrt{2}\sin \alpha &{} -\,\sqrt{2}\sin \alpha &{} \frac{\sqrt{2}}{2}\cos \alpha &{} -\,\frac{\sqrt{2}}{2}\cos \alpha &{} 0&{}0\\ \cos ^2\alpha &{} \cos ^2\alpha &{} \frac{ \sin (2\alpha )}{2} &{} -\,\frac{\sin (2\alpha )}{2} &{} 1&{}\cos (2\alpha )\\ \frac{\sin (2\alpha )}{2} &{}\frac{\sin (2\alpha )}{2} &{} -\,\frac{\cos (2\alpha )}{2} &{} \frac{\cos (2\alpha )}{2} &{} 0&{} \frac{\sin (2\alpha )}{2}\\ \sin ^2\alpha &{} \sin ^2\alpha &{} \frac{\sin (2\alpha )}{2} &{} \frac{\sin (2\alpha )}{2}&{} 1 &{}0 \end{pmatrix}=\begin{pmatrix} R_1, R_2, R_3, R_4, R_5, R_6\end{pmatrix} \end{aligned} \end{aligned}$$

associated to the eigenvalues $\lambda =(\sqrt{2},-\,\sqrt{2},\frac{\sqrt{2}}{2},-\,\frac{\sqrt{2}}{2},0,0)$. Through *P*, we can calculate $P^{-1},\;P^{1/2}$ and $P^{-1/2}$ without problems.

#### Remark 5.2

Since the system is symmetrizable, the eigenvectors are orthogonal for the quadratic form associated to *P*, i.e. for eigenvectors $r_i\ne r_j$ hold $\langle Pr_i,r_j\rangle =0$, where $\langle \cdot , \cdot \rangle$ denotes the scalar product.

#### The Boundary Conditions

The physical boundary condition follows from Maxwell’s kinetic accommodation model. We have

$$\begin{aligned} \begin{pmatrix} -\,\alpha \theta + s_xn_x+s_yn_y-\alpha R_{nn}\\ \beta t_x s_x+\beta t_ys_y+t_xn_xR_{xx}+(t_xn_y+t_yn_x)R_{xy}+t_yn_y R_{yy} \end{pmatrix}={L_{{\mathbf {n}}}} U, \qquad U=\begin{pmatrix} \theta \\ s_x \\ s_y \\ R_{xx}\\ R_{xy}\\ R_{yy} \end{pmatrix} \end{aligned}$$

with normal components $(n_x,n_y)=(\cos \gamma , \sin \gamma )$ and tangential components $(t_x,t_y)= (-\,\sin \gamma ,\; \cos \gamma )$ where $\gamma$ is the angle between the *x*-axis and the outward unit normal on $\partial \Omega$. The accommodation coefficients are given by $\alpha$ and $\beta$. We have further $R_{{{\mathbf {n}}}{{\mathbf {n}}}}= R_{xx}\cos ^2 \gamma +R_{yy} \sin ^2 (\gamma ) +2R_{xy}\cos ( \gamma ) \sin (\gamma )$ and together this gives

$$\begin{aligned} {L_{{\mathbf {n}}}}=\begin{pmatrix} -\,\alpha &{} \cos \gamma &{} \sin \gamma &{} -\,\alpha \cos ^2\gamma &{} -\,2 \alpha \cos \gamma \sin \gamma &{} -\,\alpha \sin ^2\gamma \\ 0 &{} -\,\beta \sin \gamma &{} \beta \cos \gamma &{} -\,\cos \gamma \sin \gamma &{} \cos (2 \gamma ) &{} \sin \gamma \cos \gamma \end{pmatrix}. \end{aligned}$$

Thanks to this, the boundary conditions on $\Gamma _0$ on $\Gamma _1$ reads

$$\begin{aligned} {L_{{\mathbf {n}}}} U-\begin{pmatrix} -\,\alpha \theta _0 \\ -\,u_x \sin \gamma +u_y\cos \gamma \end{pmatrix}=0 \text { on } \Gamma _0, {L_{{\mathbf {n}}}} U-\begin{pmatrix} -\,\alpha \theta _1 \\ 0 \end{pmatrix}=0 \text { on } \Gamma _1, \end{aligned}$$

where $\theta _0$ and $\theta _1$ are the prescribed temperatures on the cylinders (boundaries of $\Omega$) and $u_x, u_y$ denote the prescribed slip velocity. To simplify notations, we introduce *G* as

$$\begin{aligned} G_{{\mathbf {n}}}(x)=\left\{ \begin{array}{ll} \left( \begin{array}{l} -\,\alpha \theta _0 \\ -\,u_x \sin \gamma +u_y\cos \gamma \end{array}\right) &{}\quad \text { if } x\in \Gamma _0,\\ \begin{pmatrix} -\,\alpha \theta _1 \\ 0 \end{pmatrix}&\quad \text { if } x\in \Gamma _1. \end{array}\right. \end{aligned}$$

We follow the investigation in Sect. 4.1.2 and get to the energy balance (43)

$$\begin{aligned} \int _{\partial \Omega } \bigg ( \frac{1}{2} V^T (An_x+Bn_y) U - V^T {\Pi } {L_{{\mathbf {n}}}}U \bigg ) \partial \Omega > - \int _{\partial \Omega } V^T\Pi G_{{\mathbf {n}}}\partial \Omega . \end{aligned}$$

In our practical implementation, we look for $\Pi$ to get energy stability in the homogeneous case. Instead of working with the variable transformation $U=PV$, we select $U=P^{1/2}V$ for convenience reasons later in the implementation. Then the condition reads:

49 $$\begin{aligned} \frac{1}{2} V^T (An_x+Bn_y) U -V^T{\Pi } {L_{{\mathbf {n}}}}U= V^T \left( \left( \frac{1}{2} A_{{\mathbf {n}}}-\Pi {L_{{\mathbf {n}}}}\right) \right) P^{1/2}V >0. \end{aligned}$$

One way to achieve this is to assume that $\frac{1}{2} A_{{\mathbf {n}}}-\Pi {L_{{\mathbf {n}}}}$ has the same eigenvectors as $\frac{1}{2}A_{{\mathbf {n}}}$ and that the eigenvalues are positive, i.e. $\Pi {L_{{\mathbf {n}}}} - \frac{1}{2} A_{{\mathbf {n}}}^-$ and $\Pi {L_{{\mathbf {n}}}}$ and $\frac{1}{2} A_{{\mathbf {n}}}^-$ have the same eigenvalues.^5^ The idea behind this is that $(\frac{1}{2}A_{{\mathbf {n}}}- \Pi {L_{{\mathbf {n}}}})P^{1/2}$ is positive definite. However, this is well-defined under the condition that ${L_{{\mathbf {n}}}}P{L_{{\mathbf {n}}}}^T$ is invertible, and we obtain

50 $$\begin{aligned} {L_{{\mathbf {n}}}}P{L_{{\mathbf {n}}}}^T=\begin{pmatrix} 1+2\alpha ^2 &{} 0\\ 0&{} \frac{1}{2}+\beta ^2 \end{pmatrix}. \end{aligned}$$

This matrix is always invertible since its determinant is always positive. A solution to the problem is $\Pi {L_{{\mathbf {n}}}} P {L_{{\mathbf {n}}}}^T=R D L P^{1/2}{L_{{\mathbf {n}}}}^T$ with $D\le \frac{1}{2}\Lambda ^-$ so $\Pi = R DL P^{1/2} {L_{{\mathbf {n}}}}^T ({L_{{\mathbf {n}}}}P{L_{{\mathbf {n}}}}^T)^{-1}$ with $D\le \frac{1}{2}\Lambda ^-$ and using the transformation with $P^{1/2}$, we obtain:

$$\begin{aligned} \left( \frac{1}{2} A_{{\mathbf {n}}}-{\Pi } {L_{{\mathbf {n}}}}\right) P^{1/2}V=\lambda P^{1/2}V, \end{aligned}$$

i.e.

$$\begin{aligned} \left( \frac{1}{2} A_{{\mathbf {n}}}-\lambda I\right) P^{1/2}V=\Pi {L_{{\mathbf {n}}}}P^{1/2}V \end{aligned}$$

that is

51 $$\begin{aligned} \left( \frac{1}{2} A_{{\mathbf {n}}}-\lambda I\right) P{L_{{\mathbf {n}}}}^T({L_{{\mathbf {n}}}}P{L_{{\mathbf {n}}}}^T)^{-1}V=\Pi V \end{aligned}$$

Using *U* instead of *V* in the implementation, we have to multiply $\Pi$ with $P^{-1/2}$.

##### Remark 5.3

Another way to determine $\Pi$, we choose $\delta <0$ such that $\Pi {L_{{\mathbf {n}}}}P^{1/2}-\frac{1}{2} A_{{\mathbf {n}}}P^{1/2} =\delta \text {Id}$, and thus yields

$$\begin{aligned} \Pi =\big ( \delta P^{-1/2} +\frac{1}{2} A_{{\mathbf {n}}}\big ) {L_{{\mathbf {n}}}}^T ({L_{{\mathbf {n}}}} {L_{{\mathbf {n}}}}^T)^{-1}. \end{aligned}$$

However, this is well-defined under the condition that ${L_{{\mathbf {n}}}}{L_{{\mathbf {n}}}}^T$ is invertible. We obtain

52 $$\begin{aligned} {L_{{\mathbf {n}}}}{L_{{\mathbf {n}}}}^T=\begin{pmatrix} \frac{1}{4} (4+9\alpha ^2-\alpha ^2\cos 4\gamma )&{} -\,\frac{1}{4}\alpha \sin 4\gamma \\ -\,\frac{1}{4}\alpha \sin 4\gamma &{} \frac{1}{4} (3+4\beta ^2+\cos 4\gamma ) \end{pmatrix}. \end{aligned}$$

The matrix is always invertible since elementary calculations yield to an estimation of the determinate which can be shown to be bigger than 0.5.

#### Concrete Example

**Fig. 9:**
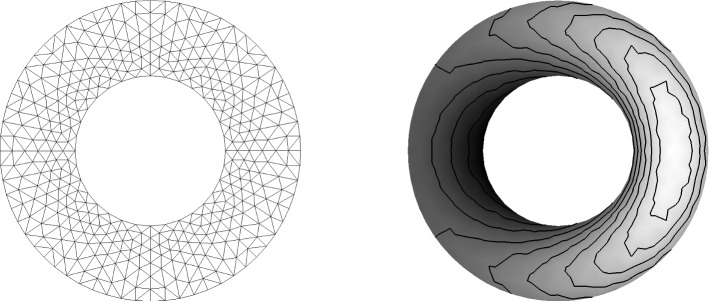
Mesh and steady state ($$t=10)$$, 3rd order scheme

We have explained how we estimate the boundary operator in the above Eq. (51). In the test, we have set the accommodation coefficients $\alpha =3.0$ and $\beta = -\,0.5$. The temperature at the boundaries are given by $\theta _0=0$ and $\theta _1=1$. Further, we have $u_x=1$ and $u_y=0$. The relaxation time is set to 0.15. Again, we use a continuous Galerkin scheme together with the above developed boundary procedure. The term $\delta$ is set to $-\,2$ and the CFL number is 0.1. We ran the problem up to steady state with a RK scheme. In Fig. 9 we show the mesh and also the result at steady state using a coarse grid. The number of triangles is 400. The problem is elliptic and smooth which cannot be seen in this first picture since the mesh is too coarse (Fig. 9).

**Fig. 10:**
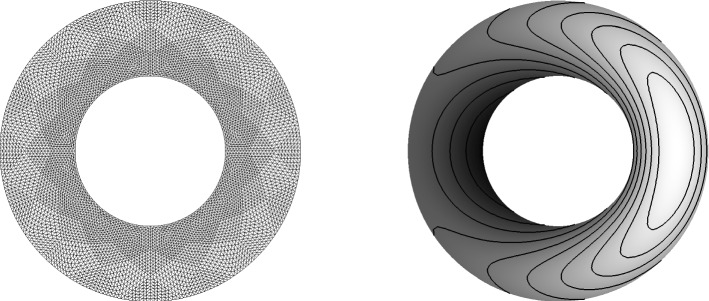
Mesh and steady state ($t=10$), 3rd order scheme

In the second test, we increase the number of elements in the mesh. Now, we are using 5824 elements and also Bernstein polynomials of second order. The mesh and the result are presented in Fig. 10. Here, we recognize the smooth behavior and the scheme remains stable only through the above described boundary procedure (Fig. 10).

## Conclusion and Outlook

In this paper, we have demonstrated that a pure continuous Galerkin scheme is stable only through the applied boundary conditions. No further stabilizations terms are needed. This contradicts the erroneous perception in the hyperbolic community about pure continuous Galerkin schemes to be unstable without additional stabilizations terms. In our approach, the application of the SAT technique is essential where we impose the boundary conditions weakly. Using this approach, we derive a suitable boundary operator from the continuous setting to guarantee that the discrete energy inequality is always fulfilled. We present a recipe on how these operators can be constructed, in detail, for scalar two-dimensional problems and two-dimensional systems. In numerical experiments, we verify our theoretical analysis. Furthermore, in one test, we demonstrate the importance of the used quadrature rule. The chosen quadrature rule in the numerical integration has to be the same as in the differential operators such that the SBP property of our Galerkin scheme is valued. If not, the Galerkin scheme suffers from stability issues. If stability can be reached only by enforcing the proper dissipative boundary conditions, there is no free meal: this procedure is very sensitive to any numerical error, like roundoff error or quadrature error. We think and hope that through our results the common opinion about continuous Galerkin and its application in CFD problems changes, modulo the restriction we have already described. This result is also interesting from a theoretical point of view, and emphasizes the positive role that the boundary conditions may have. In the companion paper [42], we consider and analyze non-linear (e.g. entropy) stability of continuous Galerkin schemes. Here, the SAT approach will also be important and some approximation for the boundary operators will be developed.

## Footnotes

1 We like to mention that also parts of the authors had this belief before starting the project.

2 Actually, the opposite is also true: Everything is a finite element scheme with a suitable quadrature rule.

3 In the finite difference community ${\underline{\underline{M}}}\,$ is called norm matrix and is classically abbreviated with *P*, c.f. [14, 27].

4 We have used simple, double and quadruple precision, the results remain the same upto machine precision.

5 Here, we denote again by − the negative eigenvalues.

## References

[1] Reed, William H., Hill, T.R.: Triangular mesh methods for the neutron transport equation. Technical Report, Los Alamos Scientific Lab., N. Mex. (USA) (1973)

[2] Cockburn B, Karniadakis GE, Shu C-W (2012). Discontinuous Galerkin Methods: Theory, Computation and Applications.

[3] Hesthaven JS, Warburton T (2002). Nodal high-order methods on unstructured grids: I. Time-domain solution of Maxwell’s equations. J. Comput. Phys..

[4] Chen T, Shu C-W (2020). Review of entropy stable discontinuous Galerkin methods for systems of conservation laws on unstructured simplex meshes. CSIAM Trans. Appl. Math..

[5] Gassner GJ (2013). A skew-symmetric discontinuous Galerkin spectral element discretization and its relation to SBP-SAT finite difference methods. SIAM J. Sci. Comput..

[6] Carpenter MH, Fisher TC, Nielsen EJ, Frankel SH (2014). Entropy stable spectral collocation schemes for the Navier–Stokes equations: discontinuous interfaces. SIAM J. Sci. Comput..

[7] Chen T, Shu C-W (2017). Entropy stable high order discontinuous Galerkin methods with suitable quadrature rules for hyperbolic conservation laws. J. Comput. Phys..

[8] Chan J (2018). On discretely entropy conservative and entropy stable discontinuous Galerkin methods. J. Comput. Phys..

[9] Kopriva DA, Gassner GJ (2014). An energy stable discontinuous Galerkin spectral element discretization for variable coefficient advection problems. SIAM J. Sci. Comput..

[10] Ranocha H, Öffner P, Sonar T (2016). Summation-by-parts operators for correction procedure via reconstruction. J. Comput. Phys..

[11] Kreiss H-O, Scherer G (1974). Finite element and finite difference methods for hyperbolic partial differential equations. Math. Asp. Finite Elem. Partial Differ. Equ..

[12] Del Rey Fernández DC, Hicken JE, Zingg DW (2014). Review of summation-by-parts operators with simultaneous approximation terms for the numerical solution of partial differential equations. Comput. Fluids.

[13] Hicken JE, Del Rey Fernaández DC, Zingg DW (2016). Multidimensional summation-by-parts operators: general theory and application to simplex elements. SIAM J. Sci. Comput..

[14] Svärd M, Nordström J (2014). Review of summation-by-parts schemes for initial-boundary-value problems. J. Comput. Phys..

[15] Abgrall R, Bacigaluppi P, Tokareva S (2019). High-order residual distribution scheme for the time-dependent Euler equations of fluid dynamics. Comput. Math. Appl..

[16] Burman E, Ern A, Fernández MA (2010). Explicit Runge–Kutta schemes and finite elements with symmetric stabilization for first-order linear pde systems. SIAM J. Numer. Anal..

[17] Burman E, Hansbo P (2004). Edge stabilization for Galerkin approximations of convection–diffusion–reaction problems. Comput. Methods Appl. Mech. Eng..

[18] Gustafsson B, Kreiss H-O, Oliger J (2013). Time Dependent Problems and Difference Methods.

[19] Thomée V, Wendroff B (1974). Convergence estimates for galerkin methods for variable coefficient initial value problems. SIAM J. Numer. Anal..

[20] Mock MS (1976). Explicit finite element schemes for first order symmetric hyperbolic systems. Numer. Math..

[21] Layton WJ (1983). Stable Galerkin methods for hyperbolic systems. SIAM J. Numer. Anal..

[22] Layton, W.J.: Stable and unstable numerical boundary conditions for Galerkin approximations to hyperbolic systems. In: Hyperbolic Partial Differential Equations, Elsevier, pp. 559–566 (1983)

[23] Gunzburger MD (1977). On the stability of Galerkin methods for initial-boundary value problems for hyperbolic systems. Math. Comput..

[24] Hicken, J.E: Entropy-stable, high-order discretizations using continuous summation-by-parts operators. In: AIAA Aviation 2019 Forum, p. 3206 (2019)

[25] Hicken JE (2020). Entropy-stable, high-order summation-by-parts discretizations without interface penalties. J. Sci. Comput..

[26] Hughes TJR, Franca LP, Mallet M (1986). A new finite element formulation for CFD: I. Symmetric forms of the compressible Euler and Navier–Stokes equations and the second law of thermodynamics. Comput. Methods Appl. Mech. Eng..

[27] Nordström J (2006). Conservative finite difference formulations, variable coefficients, energy estimates and artificial dissipation. J. Sci. Comput..

[28] Nordström J (2017). A roadmap to well posed and stable problems in computational physics. J. Sci. Comput..

[29] Carpenter MH, Gottlieb D, Abarbanel S (1994). Time-stable boundary conditions for finite-difference schemes solving hyperbolic systems methodology and application to high-order compact schemes. J. Comput. Phys..

[30] Ranocha H, Öffner P, Sonar T (2017). Extended skew-symmetric form for summation-by-parts operators and varying Jacobians. J. Comput. Phys..

[31] Öffner P, Ranocha H (2019). Error boundedness of discontinuous Galerkin methods with variable coefficients. J. Sci. Comput..

[32] Bazilevs Y, Hughes TJR (2007). Weak imposition of dirichlet boundary conditions in fluid mechanics. Comput. Fluids.

[33] Nitsche, J.: Über ein Variationsprinzip zur Lösung von Dirichlet-Problemen bei Verwendung von Teilräumen, die keinen Randbedingungen unterworfen sind. In: Abhandlungen aus dem mathematischen Seminar der Universität Hamburg, vol. 36, Springer, pp. 9–15 (1971)

[34] Kurt Otto F (1958). Symmetric positive linear differential equations. Commun. Pure Appl. Math..

[35] Ern A, Guermond J-L (2006). Discontinuous Galerkin methods for Friedrichs’ systems. I. General theory. SIAM J. Numer. Anal..

[36] Nordström J, La Cognata C (2019). Energy stable boundary conditions for the nonlinear incompressible Navier–Stokes equations. Math. Comput..

[37] Nordström J (2007). Error bounded schemes for time-dependent hyperbolic problems. SIAM J. Sci. Comput..

[38] Abgrall R (2018). A general framework to construct schemes satisfying additional conservation relations. Application to entropy conservative and entropy dissipative schemes. J. Comput. Phys..

[39] Gottlieb S, Ketcheson DI, Shu C-W (2011). Strong Stability Preserving Runge–Kutta and Multistep Time discretizations.

[40] Balay, S., Abhyankar, S., Adams, M.F., Brown, J., Brune, P., Buschelman, K., Dalcin, L., Dener, A., Eijkhout, V., Gropp, W. D., Karpeyev, D., Kaushik, D., Knepley, M. G., May, D. A., McInnes, L. C., Mills, R. T., Munson, T., Rupp, K., Sanan, P., Smith, B. F., Zampini, S., Zhang, H., Zhang, H.: PETSc Web page. https://www.mcs.anl.gov/petsc (2019)

[41] Satish B., Shrirang A., Mark, F.A., Jed, B., Peter, B., Kris, B., Lisandro, D., Alp, D., Victor, E., William, D.G., Dmitry, K., Dinesh, K., Matthew, G.K., Dave, A.M., McInnes, L.C., , Mills, R.T., Munson, T., Rupp, K., Sanan, P., Smith, B.F., Stefano, Z., Hong, Z., Hong, Z.: PETSc users manual. Technical Report ANL-95/11 - Revision 3.11, Argonne National Laboratory (2019)

[42] Abgrall, R., Nordström, J., Öffner, P., Tokareva, S.: Analysis of the SBP-SAT stabilization for finite element methods part II: entropy stability. In: Communications on Applied Mathematics and Computation (accepted) (2020) 10.1007/s10915-020-01349-z PMC7609440 33184528

[43] Abgrall, R., Meledo, E., Oeffner, P.: On the connection between residual distribution schemes and flux reconstruction. arXiv preprint arXiv:1807.01261 (2018)

[44] Abgrall, R., Öffner, P., Ranocha, H.: Reinterpretation and extension of entropy correction terms for residual distribution and discontinuous Galerkin schemes (Submitted). arXiv preprint (2019)

[45] Johnson C, Nävert U, Pitkäranta J (1984). Finite element methods for linear hyperbolic problems. Comput. Methods Appl. Mech. Eng..

[46] Torrilhon M (2016). Modeling nonequilibrium gas flow based on moment equations. Annu. Rev. Fluid Mech..

[47] Rana A, Torrilhon M, Struchtrup H (2013). A robust numerical method for the R13 equations of rarefied gas dynamics: application to lid driven cavity. J. Comput. Phys..

